# Unbalanced, Unfair, Unhappy, or Unable? Theoretical Integration of Multiple Processes Underlying the Leader Mistreatment-Employee CWB Relationship with Meta-Analytic Methods

**DOI:** 10.1177/15480518211066074

**Published:** 2021-12-27

**Authors:** Lindie H. Liang, Midori Nishioka, Rochelle Evans, Douglas J. Brown, Winny Shen, Huiwen Lian

**Affiliations:** 1 Lazaridis School of Business and Economics, 8431 Wilfrid Laurier University, 75 University Avenue West, Waterloo, ON N2L 3C5, Canada; 2 Department of Psychology, 8430 University of Waterloo, Waterloo, ON, Canada; 3 Schulich School of Business, 7991 York University, Toronto, ON, Canada; 4 Department of Management, Gatton School of Business and Economics, 4530 University of Kentucky, Lexington, KY, USA

**Keywords:** leader mistreatment, counterproductive work behaviors, social exchange, depleted self-regulatory capacity, negative affect, justice

## Abstract

Although a litany of theoretical accounts exists to explain why mistreated employees engage in counterproductive work behaviors (CWBs), little is known about whether these mechanisms are complementary or mutually exclusive, or the effect of context on their explanatory strength. To address these gaps, this meta-analytic investigation tests four theoretically-derived mechanisms simultaneously to explain the robust relationship between leader mistreatment and employee CWB: (1) a social exchange perspective, which argues that mistreated employees engage in negative reciprocal behaviors to counterbalance experienced mistreatment; (2) a justice perspective, whereby mistreated employees experience moral outrage and engage in retributive behaviors against the organization and its members; (3) a stressor-emotion perspective, which suggests that mistreated employees engage in CWBs to cope with their negative affect; and (4) a self-regulatory perspective, which proposes that mistreated employees are simply unable to inhibit undesirable behaviors. Moreover, we also examine whether the above model holds across cultures that vary on power distance. Our meta-analytic structural equation model demonstrated that all but the justice mechanism significantly mediated the relationship between leader mistreatment and employee CWBs, with negative affect emerging as the strongest explanatory mechanism in both high and low power distance cultures. Given these surprising results, as the stressor-emotion perspective is less frequently invoked in the literature, this paper highlights not only the importance of investigating multiple mechanisms together when examining the leader mistreatment-employee CWB relationship, but also the need to develop more nuanced theorizing about these mechanisms, particularly for negative affect.

Leaders exert significant influence over workgroup and organizational outcomes (Day & Lord, 1988). Unfortunately, leaders sometimes abuse their power by mistreating their followers (Foulk et al., 2018). Indeed, approximately 10% of employees experience some form of dysfunctional and untoward leader behavior, such as uncontrolled outbursts of anger, public ridicule, or credit-stealing (Tepper et al., 2017).

A significant body of research has established that when leaders mistreat their employees, these workers commonly respond by engaging in harmful actions that go against the legitimate interests of the organization—counterproductive work behaviors (CWBs; Hershcovis and Barling, 2010; Mitchell and Ambrose, 2007). CWBs have been tied to myriad negative organizational outcomes, including financial losses (Needleman, 2008), lost productivity (Taylor, 2007), and decreases in employee morale and well-being (Robinson & Greenberg, 1998). Given the significant human and organizational costs of CWBs, it is imperative to understand *why* leader mistreatment is related to employee CWBs.

To this end, several theoretical accounts have been proposed in the literature to explain why employees engage in CWBs in response to leader mistreatment. Early research has primarily drawn upon two related perspectives––a social exchange perspective (Mitchell & Ambrose, 2007; Thau & Mitchell, 2010) and a justice perspective (Tepper, 2000)––as the dominant explanations for why leader mistreatment results in employee CWBs. More recently, stressor-emotion-based arguments (Spector & Fox, 2005) and a self-regulatory capacity perspective (Thau & Mitchell, 2010) have also been advanced to explain this relationship.

Although multiple theoretical perspectives can potentially enrich our understanding of the mechanisms that may lead employees to engage in CWBs as a result of leader mistreatment, perpetually proposing and testing these mechanisms independently of each other hinders the systematic development of knowledge (Edwards, 2010; Pfeffer, 1993). This is because researchers in this literature tend to only seek confirmatory evidence for a preferred theoretical account, without testing or considering other accounts (Greenwald et al., 1986). This is problematic for the progression of theory in several ways. First, confidence in a theory that is confirmed or rejected in single studies cannot be very strong, as the findings in these primary studies may be biased due to lack of power, narrow operationalization of constructs, or sampling error (Leavitt et al., 2010; Schmidt, 1992). Second, given that there is likely to be overlap among the proposed mechanisms, testing the mediators independently does not allow us to gauge the unique effect of each mediator that is not shared with the other mediators (Fischer et al., 2017). Given these limitations, a meta-analytic integration of the literature to compare the explanatory strength and empirical overlap between these disparate theoretical accounts and their associated mechanisms that may underlie the leader mistreatment-CWB relationship is both timely and warranted.

To address these concerns, in the current paper, we conduct a meta-analytic structural equation modeling analysis (MASEM; Viswesvaran and Ones, 1995) to integrate and compare the four commonly proposed mechanisms derived from the theories mentioned above––social exchange relationship quality, interpersonal justice perceptions, state negative affect, and self-regulatory capacity impairment––to determine their respective and unique contributions in explaining the leader mistreatment-employee CWB relationship. We also seek to demonstrate whether a particular mechanism might most powerfully drive and explain this relationship.

Moreover, we recognize that not only is leader mistreatment prevalent in organizations worldwide, but also that different cultural contexts and norms may impact the absolute and relative strength of these theoretical mechanisms in explaining the relationship between leader mistreatment and employee CWB. Culture can differentially shape people’s values, attitudes, and beliefs (Vogel et al., 2015), thereby affecting how people respond to leader mistreatment. In particular, because leader mistreatment occurs in the context of a hierarchical relationship (i.e., a leader and a subordinate), the extent to which norms within a culture legitimize and maintain social hierarchy––as encapsulated by the cultural context of power distance––may affect how individuals in that culture react to leader mistreatment. Therefore, we examine the extent to which our findings can be generalized across cultures that vary in power distance.

The current paper makes several important contributions to the literature. First, by combining psychometric meta-analysis and structural equation modeling in theory testing (Viswesvaran & Ones, 1995), we simultaneously test several commonly invoked mechanisms in explaining the link between leader mistreatment and employee CWBs. Employee CWBs can be costly for organizations, and the urgency of this issue has driven diverse lines of theoretical inquiry into why employee CWBs occur in response to leader mistreatment. Unfortunately, researchers tend to favor one particular explanation for this phenomenon without testing whether the favored or novel explanation predicts above and beyond its alternatives. Thus, our research is engaging in this crucial work of comparing mechanisms for the leader mistreatment-employee CWB link both to help advance research around employee CWBs in response to leader mistreatment *and* generate practical recommendations for organizations on how best they can curb employee CWBs when leader mistreatment occurs.

Second, we pit these four theoretical accounts against one another to determine which one best explains the relationship between leader mistreatment and employee CWBs. In so doing, we heed the call for leadership scholars to model multiple mediators simultaneously (Fischer et al., 2017) and contribute to advancing knowledge for both theory and practice (e.g., identifying potential points of intervention; Van de Ven and Johnson, 2006).

Finally, our knowledge of each proposed mechanism of the leader mistreatment-employee CWB relationship can only grow by examining the impact of the cultural value of power distance on the strength of these mechanisms and how they operate. Understanding cross-cultural variation in this relationship is important, as the increasingly international reach of organizations (Lund et al., 2019) makes scholarly and practical understanding of whether, and how, these mechanisms generalize across cultures ever more pressing. Thus, our research may help organizations to understand how to break a cycle of ongoing negative exchanges between leaders and followers, particularly for organizations with teams spanning different nationalities. Below, we briefly review the literature on the relationship between leader mistreatment and employee CWBs, as well as research corresponding to each of the four mechanisms.

## Leader Mistreatment and CWBs

Leader mistreatment is defined as an overarching construct that captures a range of *active* interpersonal behavior (verbal, non-verbal, and physical) enacted by a leader directed at harming a person at work (Hershcovis, 2011). Over the past two decades, a handful of overlapping constructs related to leader mistreatment in the workplace have emerged (Hershcovis, 2011), such as abusive supervision (Tepper, 2000), supervisor social undermining (Duffy et al., 2002), supervisor incivility (Andersson & Pearson, 1999; Cortina et al., 2001), and workplace bullying (Einarsen, 2000). Although differentiating between mistreatment constructs may have utility in specific contexts, for our purposes, we do not distinguish between them. This is because, first, these mistreatment constructs all share the same conceptual definition of “[engaging] a target in a social dynamic with negative social attention and treatment in the workplace” (O'Reilly et al., 2014, p. 775). Secondly, prior research indicates that these is no sound empirical basis to do so. Specifically, a prior meta-analysis found that these constructs do not meaningfully differ in predicting employee outcomes (e.g., attitudes, well-being; Hershcovis, 2011).

Perhaps the most well-studied consequence of leader mistreatment is employee CWBs, which include discretionary and harmful work behaviors that go against the legitimate interests of the organization (i.e., employee theft, sabotage, absence from work, tardiness, and aggression directed at the supervisor, coworker, or the organization; Sackett and DeVore, 2001). In line with this view, past primary studies and meta-analyses have shown that leader mistreatment is a prominent and powerful antecedent of employee CWBs (e.g., Hershcovis & Barling, 2010; Mackey et al., 2017).

Consistent with prior literature on leader mistreatment and CWBs, we do not expect that employees only engage in *leader-directed* CWBs in reaction to leader mistreatment, given the perceived costs employees may suffer if they choose to engage in direct retaliation (e.g., supervisor-directed deviance; Lian et al., 2014b). Rather, employees typically see their leaders and co-workers as an extension of the workplace (e.g., Eisenberger et al., 2010; Shoss et al., 2013), such that they see the organization and its employees as interchangeable (Cropanzano & Mitchell, 2005). Although the average relationship between leader mistreatment and employee CWBs tends to be larger when these behaviors are directed toward leaders (*ρ*  =  .53) as opposed to coworkers (*ρ*  =  .35) or the organization (*ρ*  =  .41; Mackey et al., 2017), prior research also suggests that the relationship between leader mistreatment and CWBs directed toward these other targets could be similar to, or even exceed, leader-directed behaviors in certain contexts (80% credibility interval of *ρ* [.37, .69] for CWB directed at the leader; 80% credibility interval of *ρ* [.23, .47] for CWB directed at coworkers; 80% credibility interval of *ρ* [.24, .57] for CWB directed at the organization; Mackey et al., 2017). Thus, employees who are mistreated by their leader are prone to engaging in CWBs towards any and all possible targets (Lian et al., 2014a). Therefore, we propose that leader mistreatment positively predicts CWBs broadly.

*Hypothesis 1. Leader mistreatment is positively related to employee CWBs*.

## Mechanisms Behind Leader Mistreatment and Employee CWBs

As noted above, scholars have invoked a number of theoretical mechanisms through which leader mistreatment results in CWBs. Although these varied theoretical frameworks have contributed significantly to our understanding of why leader mistreatment leads to employee CWBs, each account has typically been tested in isolation (for an exception integrating two perspectives, see Zhang et al., 2019). The result is a chaotic theoretical landscape that leaves us with an incomplete picture as to which mechanism(s) are most central to understanding the relationship between leader mistreatment and employee CWBs, as well as whether the effects of these mechanisms hold across cultural contexts. Below, we first elaborate on each mechanism and its theoretical framework in more detail, before discussing cultural power distance and its potential impact on these mechanisms.

### Social Exchange Perspective

The social exchange perspective suggests that employees engage in CWBs in response to leader mistreatment because CWBs restore balance to their exchange relationship with their leader. Social exchange refers to a series of interactions through which parties can become dependent on one another and obligated to provide one another with non-specific favors or benefits (Colquitt et al., 2014; Cropanzano & Mitchell, 2005). By mutually fulfilling obligations, these parties attain high-quality relationships of an open-ended nature that emphasize long-term, as opposed to short-term, commitment (Blau, 1964). In mutually interdependent relationships, individuals are perceptive of, and responsive to, how others treat them in these relationships. Specifically, as posited by the norm of reciprocity (Cropanzano & Mitchell, 2005; Gouldner, 1960; Helm et al., 1972), individuals seek to “balance” harms and benefits within such a relationship. According to this norm, people will inflict harm on those who harm them (Helm et al., 1972), and evidence indicates that people who are victims of aggression are likely to counter-aggress in equal magnitude and frequency against those who aggress against them (e.g., Greco et al., 2019; Helm et al., 1972).

In the context of leader mistreatment and employee CWBs, it is well-documented that when employees experience aggression and abuse from their leader, employees’ perceptions of social exchange relationship quality (SERQ) with their leader suffers (Chang & Lyons, 2012; Xu et al., 2012). In turn, employees who perceive poor SERQ with their leader respond to this imbalance in their relationships by engaging in CWBs (Cropanzano & Mitchell, 2005; Gouldner, 1960; Helm et al., 1972). More broadly, research has shown that employees who are targets of a variety of negative treatment (e.g., distrust, Mayer et al., 2009; low ethical leadership, Scott et al., 2013) will engage in negative behaviors (Scott et al., 2013; Skarlicki & Folger, 2004).

Hypothesis 2. Social exchange relationship quality mediates the relationship between leader mistreatment and employee CWBs, such that greater leader mistreatment leads to lower social exchange relationship quality, which then leads to more employee CWBs.

### Justice Perspective

The justice perspective suggests that leader mistreatment leads to employee CWBs because mistreatment influences employees’ justice perceptions, which in turn motivates them to restore justice (Aryee et al., 2007; Mackey et al., 2017; Tepper, 2000). Employee’s interpersonal justice perceptions, the degree to which the supervisor interacts with the employee in a sensitive manner (i.e., with respect, honesty, propriety, and sensitivity; Bies and Moag, 1986; Colquitt, 2001; Greenberg, 1993), are particularly relevant in this context as it focuses specifically on employees’ perception of their encounters with their leader (vs. processes or outcomes; Holtz and Harold, 2013; Lian et al., 2012a; Tepper, 2000). Interpersonal justice perceptions are also relevant because they highlight the *moral* aspect of fairness perceptions, which may be unique to the justice perspective compared to the other three theoretical perspectives reviewed here.

The deontic model of justice (Cropanzano et al., 2003; Folger, 2001) emphasizes this moral aspect of fairness perceptions. Specifically, this model is based on the idea that individuals care about justice for its own sake. Individuals believe that—by virtue of their membership in humanity—they have the right to be treated with dignity (Rawls, 1971); to be treated otherwise is a violation of moral and social norms of conduct. When this standard is violated, individuals may seek retribution toward the transgressor (Folger et al., 2005). In fact, there is some evidence that interpersonal justice has a moral basis, and that retribution is the preferred response (Reb et al., 2006; Skarlicki et al., 2013; Skarlicki & Rupp, 2010), which may not extend to the other types of organizational justice. For example, whereas individuals prefer moral vindication (e.g., harsh punishment) as a remedy for interpersonal injustice, they may feel that monetary compensation is sufficient as a remedy for distributive injustice (Reb et al., 2006).

Supporting these arguments, past research has demonstrated that leader mistreatment is negatively associated with employee interpersonal justice perceptions (e.g., Aryee et al., 2007; Mackey et al., 2017; Tepper, 2000). That is, the more employees report being mistreated by their leader, the less they perceive that their leaders are interpersonally fair. Employee interpersonal justice perceptions, in turn, are negatively related to CWBs (e.g., Berry et al., 2007; Colquitt et al., 2001; Hershcovis et al., 2007). Since mistreatment can be viewed as a violation of moral and social norms, individuals may engage in CWBs as a form of retribution toward the transgressor (i.e., their leader). However, employees may also engage in CWBs directed at the organization or other individuals at work, because their leader is a representative of the organization and its members generally (Eisenberger et al., 2010). Indeed, meta-analytic evidence demonstrates that interpersonal justice is negatively associated with CWBs targeted at coworkers as well as at the organization (Berry et al., 2007; Colquitt et al., 2013; Hershcovis et al., 2007; Rupp et al., 2014).

We acknowledge that there are similarities between the mediating role of interpersonal justice perceptions and SERQ. That is, just as the social exchange perspective highlights employee CWBs as a form of reprisal for leader mistreatment, researchers have theorized that employees perceive leader mistreatment as unfair and thus become motivated to engage in CWBs as a form of retribution (Aryee et al., 2007; Mackey et al., 2017; Tepper, 2000). Although the two theoretical perspectives converge in that both conceptualize employee CWBs as a form of retaliation in response to leader mistreatment, the two perspectives are nonetheless distinct in key ways. Whereas an employee’s perception of SERQ focuses on balancing exchanges in a relationship and follows the norm of reciprocity, an employee’s interpersonal justice perceptions typically center on the expectations that they hold about proper treatment of individuals *in general* based on moral and social norms of conduct. As an example, based on social exchange principles, an employee who experiences mistreatment from his or her leader would not necessarily engage in CWBs if they were already “behind” in their social exchange ledger with their leader (e.g., by performing poorly despite prior leader feedback and coaching); in contrast, a justice-based perspective would predict that this same employee would likely engage in CWBs as his or her leader’s actions still violated social and moral norms of behavior.

Hypothesis 3. Interpersonal justice perceptions mediate the relationship between leader mistreatment and employee CWBs, such that greater leader mistreatment leads to lower interpersonal justice perceptions, which in turn leads to more employee CWBs.

### Stressor-Emotion Perspective

Whereas the social exchange and justice perspectives suggest that employees engage in CWBs as a form of retribution in response to leader mistreatment, the stressor-emotion perspective suggests that employees engage in CWBs as a way of coping with the negative emotions caused by the stress of leader mistreatment. The stressor-emotion perspective posits that certain workplace events are stressors (Spector & Fox, 2005), defined as demands that employees appraise as threats of a loss, particularly of material and psychological well-being (Lazarus, 1991; Lazarus & Folkman, 1984; Spector, 1988). In response, employees experience strain, which includes psychological outcomes such as negative affect (Spector, 1988). Defined as a transitory experience of unpleasant feelings, including anxiety, anger, and fear (Watson & Tellegen, 1985), state negative affect then drives employees to “self-medicate” these feelings by engaging in hedonically pleasurable counter-normative behaviors (Krischer et al., 2010; Shoss et al., 2016), including CWBs (Bushman et al., 2001; Spector & Fox, 2002).

Within the context of leader mistreatment, employees may view certain behaviors enacted by their leaders––such as inappropriate blaming, yelling, and not acknowledging their hard work––as workplace stressors. These behaviors serve as stressors, because employees are likely to appraise these leader behaviors as threats to their well-being (Yagil et al., 2011; Zhou et al., 2015). Specifically, being a target of leader mistreatment signals to employees that they are not deserving of respectful treatment (Lian et al., 2012a), that they are powerless (Deci & Cascio, 1972; Duffy et al., 2002; Semmer et al., 2005), and that they lack job competence (Lian et al., 2012a). Essentially, these employees experience threatened belongingness, autonomy, and competence needs, respectively (Aquino & Thau, 2009; Lian et al., 2012a).

In turn, the stress associated with leader mistreatment can cause employees to experience strain, of which negative affect serves as an indicator. Demeaning behaviors––which breaches one’s ideas about the treatment one deserves from others (Folger, 1987)––can be viewed as an offence or a provocation that elicits negative emotional states (Lazarus, 1991; Lian et al., 2014a; Mayer et al., 2012). Moreover, mistreatment from the leader (e.g., verbal attacks and angry gestures) can be “emotionally traumatic” for employees (Harvey & Keashly, 2005; Restubog et al., 2011, p. 715), and is associated with psychological distress and negative emotions (e.g., anxiety, fear, and depression; Bowling and Beehr, 2006; Selye, 1974). In short, a large body of research finds that employees experience a broad range of negative emotions in response to leader mistreatment.

As experiencing negative affect is uncomfortable and distressing, employees may seek ways to cope with their negative emotions (Larsen, 2000). One way in which employees may cope with negative affect is by engaging in CWBs. This is because employees are hedonically inclined to engage in negative actions to experience pleasure (Spector & Fox, 2002). Active forms of CWBs, such as theft and sabotage, may be seen as a way to improve one’s affect. Indeed, many individuals believe that venting their anger will help them feel better (Bushman et al., 2001). In particular, when people are able to inflict harm on a transgressor, not only do they expect to feel pleasure, or catharsis (Knutson, 2004), but areas of the brain related to feelings of pleasure are also activated (e.g., caudate nucleus; de Quervain et al., 2004). Other research has found that employees have better emotional outcomes when they perform CWBs in response to stress (Krischer et al., 2010). Additionally, passive forms of CWBs, such as withdrawing from work tasks, can also serve as an emotion-focused coping strategy, whereby employees remove themselves from distressing situations at work or attempt to prevent further mistreatment (Krischer et al., 2010). For example, leader mistreatment is related to employee withdrawal, such that employees who are mistreated tend to avoid interaction with their supervisors (Whitman et al., 2014) and distort truths to avoid facing problems (Tepper et al., 2007).

Overall, the stressor-emotion perspective offers a different motivation for why mistreated employees engage in CWBs as a result of leader mistreatment. In contrast to the retribution-based accounts offered by the social exchange or justice perspectives, which suggest that employees engage in CWBs primarily as a tit-for-tat response against a target that they perceive to have transgressed against them in some way (i.e., my leader does something to harm me, so I should do something to harm him or her back), the stressor-emotion perspective positions CWBs as manifestations of employees’ desires and attempts to directly regulate their own emotions.

Hypothesis 4. State negative affect mediates the relationship between leader mistreatment and employee CWBs, such that greater leader mistreatment leads to more employee state negative affect, which in turn leads to more employee CWBs.

### Self-Regulatory Capacity Perspective

The self-regulatory capacity perspective suggests that when employees are being mistreated at work, they become unable to inhibit subsequent CWBs, as mistreatment impairs their self-regulatory capacity to regulate their behaviors (Thau & Mitchell, 2010). A self-regulation framework (Kotabe & Hofmann, 2015) proposes that exerting self-control requires individuals to override their immediate desires (e.g., lashing out at someone who mistreated you, spending time on social media as opposed to working) that are in conflict with a higher-order goal (e.g., adhering to the societal norms of appropriate conduct in the workplace; Liang et al., 2018a; Metcalfe and Mischel, 1999). When a desire conflicts with a higher-order goal, individuals initiate the self-control process to inhibit acting impulsively. All else being equal, the greater the amount or degree of self-regulatory capacity––“cognitive resources in a given moment to override desire with a higher order goal” (Kotabe & Hofmann, 2015, p. 625)––that people have, the more effective their inhibition (Kotabe & Hofmann, 2015). In contrast, when self-regulatory capacity is impaired, individuals tend to disregard the long-term implications of their behavior in favor of fulfilling immediate desires, such as engaging in unethical behaviors (Gino et al., 2011), lashing out at someone who frustrates goal attainment (Liang et al., 2016), or retaliating against someone who mistreats them (Lian et al., 2014a).

Experiencing mistreatment from a leader impairs employees’ self-regulatory capacity for several reasons. First, mistreatment is “psychologically challenging” (Thau & Mitchell, 2010, p. 1011) in the sense that employees expend resources in sense-making (Rosen et al., 2016) and rumination (Liang et al., 2018b) to understand the mistreatment (Thau & Mitchell, 2010). Second, mistreated employees expend cognitive resources to formulate an appropriate reaction to the mistreatment (Rosen et al., 2016), which, ironically, weakens their capacity to override impulsivity (Baumeister & Heatherton, 1996; DeWall et al., 2007), as demonstrated by growing evidence of impaired self-regulatory capacity following mistreatment (Thau & Mitchell, 2010).

Although the self-regulation literature would not predict specifically that individuals engage in CWBs as a result of impaired self-regulatory capacity, CWBs may nevertheless be the most likely response in the context of workplace leader mistreatment. This is because leader mistreatment is an interpersonal provocation that elicits a desire in employees to engage in immediate, gratifying behaviors that are ultimately counterproductive to their long-term goals, which include a number of CWBs (e.g., theft, interpersonal aggression, withdrawal, and absenteeism; Sackett and DeVore, 2001). Such behaviors are automatic, well-learned, and effortless (MacDonald, 2008), resulting in satisfaction and pleasure (de Quervain et al., 2004).

In addition, giving into one’s baser desires and engaging in CWBs can be regarded as a form of self-control failure, since CWBs violate the higher-order goal of following established social norms of maintaining workplace civility (Bennett & Robinson, 2000; Thau & Mitchell, 2010) and are frowned upon by peers and supervisors (Lian et al., 2012a; Lievens et al., 2008; Rotundo & Sackett, 2002). In fact, when cognitive and attentional resources are impaired—as occasioned by leader mistreatment—people are more likely to both disregard goals of engaging in socially appropriate conduct and succumb to their desires to enact CWBs for immediate gratification (Lian et al., 2017). Specifically, one’s sense of self-regulatory capacity impairment has been shown to relate to behaviors that are considered CWBs, such as withdrawal (Sliter et al., 2012), cyberloafing (Wagner et al., 2012), and workplace deviance (Christian & Ellis, 2011).

It is important to note that this perspective offers a unique explanation of the leader mistreatment-CWB link from the three previously discussed perspectives, which frame engaging in CWBs as goal-directed behaviors (i.e., for the purpose of reciprocity, moral retribution, or coping, respectively). On the other hand, the self-regulatory capacity perspective argues that engaging in CWBs in response to leader mistreatment is not goal-directed, but a “side-effect” of employees’ impaired cognitive and attentional resources that are needed to inhibit certain drives and behaviors.

Hypothesis 5. Employee’s impaired self-regulatory capacity mediates the relationship between leader mistreatment and employee CWBs, such that greater leader mistreatment leads to greater impairment in self-regulatory capacity, which in turn leads to more employee CWBs.

### Non-Shared Predictive Power of the Proposed Mediators

As stated earlier, our main aim in the current study was to synthesize and compare disparate theoretical accounts of why leader mistreatment at work leads to CWBs. Specifically, our review of the literature suggests that there are four main reasons offered for why leader mistreatment results in employee CWBs: (1) tit-for-tat social exchange of negative actions (i.e., social exchange perspective), (2) retaliation for the leader’s violation of social and moral standards (i.e., justice perspective), (3) as a way to manage or cope with the negative emotional reactions that result from stress (i.e., stressor-emotion perspective), and (4) inability to suppress impulsive behaviors as the result of lack of self-regulatory resources (i.e., self-regulatory capacity perspective). Although the first two perspectives are featured most prominently in the literature in explaining the leader mistreatment and follower CWBs relationship, all four perspectives reviewed above are theoretically plausible and each has received some empirical support when examined individually. However, there is a strong likelihood that the proposed mechanisms overlap with one another to a non-trivial extent (e.g., self-regulatory impairment is likely accompanied by state negative affect, an imbalanced social exchange relationship may be associated with negative emotions). As such, previous research testing each mechanism independently without considering their shared variance gives limited insight into the non-shared and unique predictive power of each mechanism. As such, we do not make specific predictions about which of the four proposed mediators has the strongest explanatory power or which mechanism(s) stands out as explaining the most non-shared or unique variance. Instead, we examine the following exploratory research question:

Research Question 1: Which mechanism (i.e., social exchange relationship quality, interpersonal justice perceptions, state negative affect, or impaired self-regulatory capacity) most strongly explains the relationship between leader mistreatment and employee CWBs?

### The Moderating Effects of National/Cultural Power Distance

Both theory (e.g., Martinko, Harvey, Brees, & Mackey, 2013; Tepper, 2007; Tepper et al., 2017) and empirical evidence (e.g., Vogel et al., 2015) suggest that employee reactions to leader mistreatment vary depending on the culture’s power distance, defined as the extent to which individuals accept and expect that power is unequally distributed among members of organizations and institutions (Hofstede, 2011). In particular, individuals are less likely to perceive leader mistreatment as a harmful or violating act in high power distance cultures (relative to low power distance cultures) because individuals in high power distance cultures are more likely to view exploitive and hostile behaviors of leaders as legitimate ways to control and influence subordinates, compared to individuals in low power distance cultures (Li & Cropanzano, 2009; Tyler et al., 2000; Vogel et al., 2015). Consequently, employees might be less likely to experience changes in their SERQ or interpersonal justice perceptions as a result of leader mistreatment, ultimately leading to less employee retaliation in the form of CWBs. Empirical studies have shown some support for this claim; abusive supervision has a weaker effect on individuals’ perceptions of interpersonal justice in high compared to low power distance cultures (Lian et al., 2012b; Vogel et al., 2015; Wang et al., 2012). As such, we expect that the mediating effects of social exchange relationship quality (SERQ) and interpersonal justice are weaker in high power distance cultures, compared to low power distance cultures.

The influence of cultural power distance on the mediating effects of negative affect and self-regulatory capacity impairment are less clear. There is some evidence that power distance dampens the impact of leader mistreatment on employee well-being (Lin et al., 2013), therefore, it is possible that individuals in high power distance cultures might be less likely to cope with their emotions by behaviorally expressing their emotions, such as through CWBs. As for self-regulatory capacity impairment, we speculate that individuals in high power distance cultures might be less likely to perceive leader mistreatment as a difficult experience, which in turn means that they might be less likely to experience self-regulatory capacity impairment as a result. Consequently, the mediating effects of negative affect and self-regulatory capacity impairment may be weaker in high relative to low power distance cultures.

Empirically, we examine cultural power distance as a moderator in following ways. First, following *Research Question 1* (i.e., which mechanism most strongly explains the mistreatment-CWB relationship?) we examine the extent to which the strength of the mediators, *relative to one another*, vary across cultures. That is, we ask, does the strongest mechanism consistently remain the strongest when examined across cultures that vary in power distance?

Research Question 2: Which mechanism (i.e., social exchange relationship quality, interpersonal justice perceptions, state negative affect, or impaired self-regulatory capacity) most strongly explains the relationship between leader mistreatment and employee CWBs across cultures varying in power distance?

In addition, we test the moderating effects of power distance on the four proposed mechanisms. That is, we examine whether the strength of each mediated effect differs across cultures that vary on power distance.

Research Question 3: Will the mediating effects of social exchange relationship quality, interpersonal justice perceptions, state negative affect, and impaired self-regulatory capacity of the leader mistreatment and CWBs relationship become weaker or stronger across cultures varying in power distance?

## Method

### Data Collection

**Literature search*.*** We conducted our literature search in Web of Science (1900–2017) on October 2017. To compile search terms, we referenced past published meta-analyses in this domain (e.g., Berry et al., 2012; Colquitt et al., 2013; Joseph et al., 2015; Mackey et al., 2017; Schyns & Schilling, 2013) and reviewed commonly used terms for the constructs of interest. For interpersonal mistreatment, several terms were taken from Schyns and Schilling (2013) and Bowling and Beehr (2006). Additionally, we used the same terms as Berry et al. (2012) for uncovering work on counterproductive work behavior, but added the acronym “CWB”.

With regards to proposed mediating mechanisms, for interpersonal justice perceptions, we conducted a broad search using various justice terms and types (e.g., workplace justice, distributive justice, interpersonal justice, organizational fairness), while referencing terms such as “workplace” and “organization” as descriptors. We conducted a broad search because this literature search was part of a larger project in which various indicators of justice was of interest. For SERQ, various indicators are used in the literature, with common examples being SERQ (Bernerth et al., 2007; Colquitt et al., 2014; Shore et al., 2006), trust, leader-member exchange (LMX), perceived organizational support (POS), and affective commitment (Colquitt et al., 2014; Cropanzano & Byrne, 2000). Thus, terms referring to these variables were included as search terms. For state negative affect, we searched for relevant studies using terms such as negative affect, negative mood, and negative emotion. Finally, for self-regulatory capacity impairment, we extracted common terms describing relevant variables from the literature, such as self-control, depletion, fatigue, and exhaustion (Cordes & Dougherty, 1993; Sonnentag & Zijlstra, 2006; Tangney et al., 2004). A complete list of search terms can be found in Appendix A.

To further target articles that included estimates of relationships (i.e., correlations) between the constructs of interest, we conducted our search by systematically combining search terms for two constructs at a time, using the “or” function between search terms within a construct (e.g., “mistreatment” or “abusive supervision”), and combining these terms with search terms for another construct (e.g., “social exchange”) using the “and” function. We specifically searched for workplace-related literature using the “and” function and adding terms such as “organization” and “work”. We excluded documents that were not in English, and we limited our search to return “article” or “review” only. In total, the search yielded 11,319 articles. Once all duplicate records were removed, 9,431 articles remained.

The broad search strategy captured many articles that are outside of our field (e.g., clinical psychology, entrepreneurship, and marketing), which is problematic because those articles are likely to be irrelevant in terms of the phenomenon that we are interested in examining. We thus further screened each article for the journal in which it was published, a strategy employed by previous meta-analyses (e.g., Colquitt et al., 2000). To select an appropriate set of journals, we first identified six meta-analyses that were recently published in journals within the organizational sciences/management field that are related to the topic of our current meta-analysis (i.e., Berry et al., 2012; Colquitt et al., 2001; Hershcovis and Barling, 2010; Mackey et al., 2017; Park et al., 2019; Schyns and Schilling, 2013). Using the references section of these meta-analyses, we counted the number of times each journal is included across these meta-analyses. Based on the journal count information, we compiled a list of journals that represent approximately 80% of the total number of articles included in the aforementioned meta-analyses. Each journal in this list were cited more than 3 times across these meta-analyses. Two journals in social psychology (i.e., *Journal of Personality and Social Psychology* and *Journal of Experimental Social Psychology*) were excluded from our journal list, because most papers published in those journals are outside the workplace context. Journals that primarily publish review papers were not considered (e.g., *Academy of Management Review*), as primary research reports are needed for our analyses. The complete list of journals that were considered can be found in Appendix B. We selected articles that were published within this journal list. After further selecting by journals, 1,335 records were retained for screening.

We screened the studies hierarchically (i.e., if a study did not meet an earlier criterion, it was not considered for subsequent criteria). First, studies were excluded if they were qualitative studies, meta-analyses, or narrative reviews. To generalize our findings to organizations, we then excluded studies if the samples were not working individuals (e.g., non-working students). The article had to report at least one effect size for the relationship between any two constructs in our model. We also excluded studies in which the relationship of interest was part of or occurred after experimental manipulations, because we are interested in leader mistreatment as it naturally occurs in the workplace. The first three authors screened 20 articles independently to assess agreement, and they agreed on which articles should be included 75% of the time. After resolving the initial screening discrepancy, the three authors agreed 100% on which articles should be included in a subsequent set of 20 articles. Subsequently, the first three authors screened studies independently. After this process, 163 eligible articles were identified.

***Additional data sources.*** Based on Chambless and Hollon’s (1998) recommendation, we use *k*  =  3 as the minimum number of primary studies for each meta-analytic correlation. After coding the studies identified from our search process, there were several links in the correlation matrix with less than three primary studies (*k*  =  1 for justice and self-regulation impairment, *k*  =  1 for social exchange directed at supervisor and CWB directed at supervisor, and *k*  =  1 for self-regulation impairment and CWB directed at organization). Thus, we took the following steps to ensure that we have at least three studies for each cell in the correlation matrix. First, we sent out a call to Academy of Management and Society for Industrial and Organizational Psychology listservs for unpublished data for intercorrelations between all variables in our meta-analysis. We received two responses; after screening for eligibility, we coded two papers representing three studies. Second, we screened our own unpublished data for intercorrelations between all relevant variables in our meta-analysis and coded two of our own unpublished studies.

Unfortunately, after this process, some cells still fell below this *k*  =  3 threshold. Therefore, in line with past meta-analyses (e.g., Shockley et al., 2017), we collected an independent sample (*N*  =  238) of full-time employees with participants recruited from Amazon’s Mechanical Turk. Participants in this sample (65% female; Age *M*  =  33.69, *SD*  =  8.43) had been employed in their current organization for an average of 6.58 years (*SD*  =  5.88), and 57.7% of employees held a managerial position. Specifically, we used Tepper’s (2000) abusive supervision scale to assess leader mistreatment (*α*  =  .97), McAllister’s (1995) affect-based trust scale for social exchange quality directed at the supervisor (*α*  =  .94), Meyer et al.’s (1993) affective commitment scale for social exchange quality directed at the organization (*α*  =  .88), Colquitt’s (2001) scale for interpersonal justice perceptions (*α*  =  .91), Maslach et al.’s (1996) emotional exhaustion scale for state self-regulatory impairment (*α*  =  .95), Watson and Clark’s (1999) PANAS-X scale for state negative affect (*α*  =  .96), Mitchell and Ambrose’s (2007) supervisor-directed deviance scale for CWB directed at supervisor (*α*  =  .97), and Bennett and Robinson’s (2000) organizational deviance scale for CWB directed at the organization (*α*  =  .95). For the main or overall meta-analytic analyses, we used composite correlations of the two target-specific social exchange quality and CWB scales.

**Figure 1.**
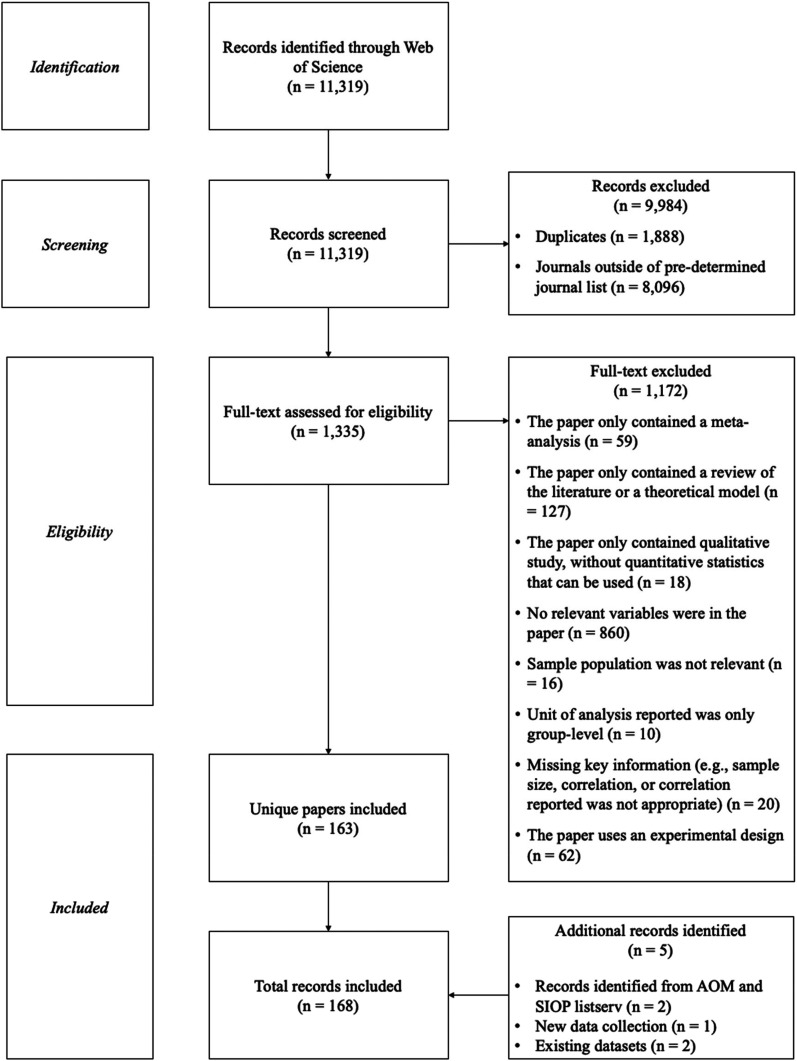
Flow chart of literature search and screening.

A flow diagram summarizing the process of literature search, screening, and data collection can be found in Figure 1. The above search and collection of data resulted in five additional studies. Combined with the literature search, our final number of records included in the meta-analysis was 168, from which we extracted 214 studies (i.e., 214 independent samples).

### Coding of Variables

***Leader mistreatment.*** As described above, we define leader mistreatment as *active* interpersonal behaviors (verbal, non-verbal, and physical) enacted by a leader directed at harming a person at work (Hershcovis, 2011). We thus included various indicators of leader mistreatment, including abusive supervision (Tepper, 2000), supervisor social undermining (Duffy et al., 2002), supervisor incivility (Andersson & Pearson, 1999; Cortina et al., 2001), and workplace bullying (Einarsen, 2000).

We excluded a number of indicators that did not meet this definition. First, we excluded measures of ostracism (i.e., experience of social disengagement through lack of attention and treatment), as prior research suggest that this is a qualitatively different experience with different consequences than mistreatment (e.g., Ferris et al., 2017; O'Reilly et al., 2014). Second, we also excluded measures of workplace conflict (e.g., Jockin et al., 2001), which typically assess how often the focal employee and the supervisor argue or have disagreements. It was excluded because conflict involves bidirectional exchanges, and unlike leader mistreatment, the focal employee is not necessarily harmed by the leader. Third, we excluded measures where the source of the mistreatment was not the focal employees’ supervisor (e.g., coworker incivility), or the source could not be identified based on the descriptions provided by the authors. Finally, some studies used measures of justice perceptions as proxy for mistreatment. However, we did not code these variables as mistreatment given that the justice perspective argues that justice perceptions *underlie* the relationship between mistreatment and outcomes, but (in)justice is not necessarily mistreatment (Tepper, 2000).

***CWB.*** We define CWBs as counter-normative voluntary behaviors initiated by employees that go against the legitimate interests of the organization (Bennett & Robinson, 2000; Dalal, 2005; Sackett, 2002; Sackett & DeVore, 2001). A range of behaviors, including theft, sabotage, and dishonest practices, were included in our analyses. We included withdrawal behavior, as it is conceptually consistent with the definition of CWB (Berry et al., 2012; Spector et al., 2006) and exhibits similar empirical relationships with correlates (Carpenter & Berry, 2017). However, we excluded withdrawal measures that were purely psychological in nature (e.g., “thought about being absent”) to focus on behaviorally-oriented measures (e.g., “let others do my job”). Finally, we excluded accidents or other damaging behaviors that are likely to be unintentional.

***SERQ***. Historically, researchers have operationalized SERQ in many different ways. These operationalizations have been strongly influenced by Cropanzano and Byrne’s (2000) speculations about the appropriate indicators of SERQ (i.e., affective commitment, trust, perceived support, contract breach, and exchange quality). However, more recent research has demonstrated that some common SERQ indicators are not content valid representations of the construct (e.g., POS and perceived contract breach), as many purported indicators actually only focus on antecedents to (e.g., benefits) or consequences of (e.g., reciprocation) SERQ, rather than SERQ per se (Colquitt et al., 2014). Thus, based upon recommendations by Colquitt et al. (2014), we included the following constructs as content-valid indicators of SERQ: affect-based trust, affective commitment, SERQ-specific indicators, leader member exchange (LMX), and willingness to be vulnerable.

***Interpersonal justice perceptions***. We operationalized interpersonal justice as employees’ perceptions of the degree to which their supervisor interacts with them in a sensitive, respectful, and polite manner (Greenberg, 1993). The most widely used measure in the literature currently is Colquitt’s (2001) four-item scale. However, prior to the publication of Colquitt’s (2001) measure, researchers used a variety of measures; some researchers combined measures of informational justice (e.g., clarity of explanations) or procedural justice (e.g., adherence to policies) with interpersonal justice in their studies (e.g., Niehoff and Moorman, 1993). Because we argue that interpersonal justice perceptions are the most relevant in the context of leader mistreatment and employee CWBs, we exclude measures that include other forms of justice.

As we are interested in examining interpersonal justice perceptions as a mechanism explaining the relationship between leader mistreatment and employee CWBs, we argue that it is most appropriate to focus on interpersonal justice perceptions that concern the employee’s *leader*. That is, we exclude studies from our analyses that do not explicitly state that the employees were instructed to think of their leader(s) when responding to the items and those that refer to other referents. Finally, consistent with the other mechanisms discussed in this paper, interpersonal justice perceptions are subjective perceptions that employees hold. Thus, we only included measures of interpersonal justice perceptions reported by focal employees.

***State negative affect***. We operationalized state negative affect as a transitory experience of distressing mood states (Colquitt et al., 2013). As such, we include both general indicators of negative mood, typically measured by the PANAS (Watson & Clark, 1999), as well as indicators of specific negative emotions (e.g., anger, disgust, and contempt). We only included measures that assessed state, and not trait, negative affect. Trait affect refers to people’s general disposition to feel particular emotions across situations (Colquitt et al., 2013) and, thus, do not conceptually represent an outcome resulting from events, such as mistreatment at work. We made the trait-state distinction by examining the scale instruction or item stem. A measure was categorized as a state measure only if the instruction referred to a specific time frame or to a specific relationship or context. Most studies included in our analysis asked participants to report their affect or emotions over the past week(s), past month(s), at work, or directed at a target (e.g., supervisor).

***Self-regulatory capacity impairment***. Self-regulatory capacity impairment was defined as a subjective experience characterized by lacking in cognitive, attentional, or mental resources and feeling unable to adequately perform tasks, inhibit impulses and desires, and override one’s dominant course of behavior. In addition to measures that were explicitly created to capture this construct (e.g., Ciarocco et al., 2007), we also included other indicators, such as fatigue and emotional exhaustion (e.g., Maslach and Jackson, 1981). Subjective experiences of fatigue and emotional exhaustion are theoretically and empirically consistent with the concept of impaired self-regulatory capacity (Hagger et al., 2010; Inzlicht et al., 2014) and these measures are very similar to measures of impaired self-regulatory capacity (e.g., “I feel emotionally drained from my work” and “I feel drained”). Measures of burnout were excluded if they included other dimensions, such as cynicism and inefficacy (Maslach et al., 2001). Measures were also excluded if impaired self-regulatory capacity outcomes (e.g., tardiness and rumination) or antecedents (e.g., sleep quality and leadership behavior; Lian et al., 2017) were used as proxies.

***Regional origin of the sample.*** To answer *Research Questions 2* and *3*, for each study, we recorded the region (e.g., country) in which the sample was collected. We then imputed Hofstede’s cultural dimension scores for each sample. The scores range from 0 to 100 and were obtained from https://geerthofstede.com (Hofstede, 2015) in 2018. Samples for which the article did not report the geographic origin (36%) or reported a region that did not have corresponding cultural dimension scores (7%) were treated as missing data and were omitted from the moderation analyses.

***Time lags between measurements.*** In some studies, measures were separated by time. To minimize variation across samples due to different time lags between measurements and to have a consistent rule across coders, we coded the correlation between variables measured at the earliest time in the study and with the shortest time lag between them. For example, for the longitudinal study by Lapointe et al. (2011), we coded the correlation between affective commitment (an SERQ variable) and emotional exhaustion (a self-regulatory capacity impairment variable) both measured at Time 1. As such, all the reported results are derived from cross-sectional measurements or variables measured with minimal time lags (see our supplemental analyses for a separate analysis of time-lagged measurements)^1^.

### Coder Training and Coding Process

Prior to coding, the first three authors coded 20 articles independently to assess the reliability of their coding. Following Colquitt et al. (2013), we computed the *ICC*(2) index, which reflects agreement among different judges regarding a rated variable (Bliese, 2000). The information coded from articles (i.e., identification of variables of interest, reliabilities, effect sizes, and sample sizes) had an *ICC*(2) of .89 (95% CI [.793, .944], *F*(18, 58)  =  8.93, *p* < .001, indicating adequate interrater reliability (Bliese, 2000). We also took this opportunity to clarify our coding procedure and resolve any issues. After resolving disagreements through discussion, the three authors each coded a different subset of articles, and any concerns raised during coding were discussed among these three authors until consensus was reached. This step was taken to ensure that the coding criteria and process were clear to all coders.

### Data Analyses

***Meta-analyses.*** We used Hunter and Schmidt’s (2004) random effects meta-analysis method, correcting for sampling error and measurement error in the predictor and criterion measures. As most studies in our database reported reliability information, we corrected each study individually for unreliability. For variables in which reliability estimates were not reported, we imputed the average of all other reliability estimates for that construct, in line with prior research (Colquitt et al., 2013). The number of studies with imputed reliabilities were as follows: *k*  =  2 for interpersonal mistreatment, *k*  =  1 for social exchange relationship quality, *k*  =  3 for interpersonal justice, *k*  =  2 for impaired self-regulatory capacity, *k*  =  4 for negative affect, and *k*  =  6 for CWBs. Correcting for unreliability due to transient error and scale-specific error is conceptually important for this study (e.g., mood of the participant when reporting mistreatment); however, given that most studies only reported Cronbach’s alpha, we were limited to using the reliability estimate for corrections in the predictor and criterion measures.

Whenever a study reported effect sizes for multiple indicators of the same construct (e.g., support, trust, and commitment), we computed an equally weighted composite as well as the reliability of the composite variable using the formulas provided by Hunter and Schmidt (2004, pp. 435–438). The new effect size and reliability associated with the composite was then used in the meta-analysis, as recommended by Viswesvaran and Ones (1995).

***Mediation analyses.*** To estimate our proposed model, we used MASEM (Viswesvaran & Ones, 1995). We first constructed a meta-analytic correlation matrix. We then computed the harmonic *N* following the formula provided by Colquitt et al. (2000): *k*/(1/*N*_1_ + 1/*N*_2_ + … + 1/*N_k_*), where *k* refers to the number of study correlations (i.e., number of cells in the matrix) and *N* refers to the sample sizes of each study. Harmonic *N* was used as the sample size for the matrix because it is more conservative than the arithmetic *N* (Viswesvaran & Ones, 1995). We tested our theorized model with the SEM software Mplus 7.4 (Muthén & Muthén, 1998-2012), whereby we ran two separate models. Specifically, the direct effect of mistreatment to CWB (*Hypothesis 1*) was tested by regressing leader mistreatment on CWB without any mediators in the model, and the mediation hypotheses (*Hypothesis 2*, *3*, *4*, and *5*) were tested in a separate SEM model where we regressed leader mistreatment on CWB with all mediators simultaneously (i.e., SERQ, interpersonal justice perceptions, impaired self-regulatory capacity, and state negative affect). The statistical significance of the indirect effects was determined using 95% confidence intervals.

***Moderation analyses.*** To answer our research questions regarding the moderating effect of culture on the proposed mediational pathways, we conducted subgroup analyses (Schmidt & Hunter, 2015) by splitting the coded data at a score of 50 on Hofstede’s cultural dimension scores, in line with prior meta-analytic studies (e.g., Rockstuhl et al., 2012). Samples with scores less than 50 were coded as low power distance, and samples with scores higher than 50 were coded as higher on power distance. We chose to dichotomize power distance because this more faithfully represents the distribution of study locations in our dataset, as many studies were either from the West (e.g., United States) or the East (e.g., China)^2^. The same meta-analytic procedure described above was then used to compute the meta-analytic estimates and mediation analyses within each subgroup. We compared the strength of the indirect effects across subgroups as evidence for moderation. Unfortunately, interpersonal justice could not be examined as a mediator in these moderation analyses, because the necessary correlations were not available for one subgroup (i.e., the correlation between leader mistreatment and interpersonal justice for the high power distance subgroup).

## Results

The meta-analytic correlation matrix is presented in Table 1. These bivariate results indicate that, consistent with *Hypothesis 1*, leader mistreatment was positively and strongly correlated with employee CWBs (*ρ*  =  .51, *k*  =  50, 95% CI [.464, .556]). Additionally, leader mistreatment was associated with each proposed mediator in the expected direction, with the strongest relationship found between leader mistreatment and interpersonal justice perceptions (*ρ*  =  −.64, *k*  =  9, 95% CI [ − .679, − .592]), followed by state negative affect (*ρ*  =  .53, *k*  =  16, 95% CI [.458, .600]), and finally, more moderate relationships with both self-regulatory capacity impairment (*ρ*  =  .37, *k*  =  19, 95% CI [.329, .413]) and SERQ (*ρ*  =  −.36, *k*  =  36, 95% CI [ − .419, − .295]). Further, each of the proposed mediators predicted CWBs as theorized; the strongest relationship was found for state negative affect (*ρ*  =  .49, *k*  =  35, 95% CI [.428, .600]), followed by more modest and similar in magnitude relationships with interpersonal justice (*ρ*  =  −.31, *k*  =  19, 95% CI [ − .384, − .242]), self-regulatory capacity impairment (ρ  =  .24, *k*  =  14, 95% CI [.181, .308]), and SERQ (ρ  =  −.23, *k*  =  21, 95% CI [ − .297, − .168]), respectively.

**Table 1.** Meta-Analytic Correlation Matrix.

Variable			1	2	3	4	5	6
1.	Leader mistreatment	*k* (*N*)	-					
		*r* (*SD_r_*)	-					
		*ρ* (*SD*_ρ_)	-					
		CI	-					
		CV	-					
2.	Social exchange relationship	*k* (*N*)	36 (12477)	-				
	quality (SERQ)	*r* (*SD_r_*)	−.30 (.16)	-				
		*ρ* (*SD*_ρ_)	−.36 (.18)	-				
		CI	[ − .42, − .30]	-				
		CV	[ − .66, − .06]	-				
3.	Interpersonal justice perceptions	*k* (*N*)	9 (2843)	26 (19610)	-			
		*r* (*SD_r_*)	−.59 (.06)	.48 (.16)	-			
		*ρ* (*SD*_ρ_)	−.64 (.05)	.55 (.19)	-			
		CI	[ − .68, − .59]	[.48, .63]	-			
		CV	[ − .73, − .55]	[.25, .86]	-			
4.	State Negative affect	*k* (*N*)	16 (4831)	13 (3041)	9 (2073)	-		
		*r* (*SD_r_*)	.45 (.14)	−.24 (.14)	−.31 (.11)	-		
		*ρ* (*SD*_ρ_)	.53 (.13)	−.30 (.15)	−.35 (.11)	-		
		CI	[.46, .60]	[ − .39, − .21]	[ − .43, − .26]	-		
		CV	[.31, .75]	[ − .53, − .06]	[ − .53, − .17]	-		
5.	Self-regulatory capacity	*k* (*N*)	19 (6286)	39 (10575)	4 (1074)	15 (4614)	-	
	impairment	*r* (*SD_r_*)	.33 (.09)	−.35 (.10)	−.31 (.12)	.51 (.10)	-	
		*ρ* (*SD*_ρ_)	.37 (.08)	−.43 (.10)	−.35 (.14)	.60 (.11)	-	
		CI	[.33, .41]	[ − .47, − .40]	[ − .49, − .20]	[.54, .66]	-	
		CV	[.25, .50]	[ − .59, − .27]	[ − .57, − .12]	[.42, .79]	-	
6.	CWB	*k* (*N*)	50 (16167)	21 (7812)	19 (5188)	35 (8618)	14 (4261)	-
		*r* (*SD_r_*)	.44 (.15)	−.19 (.11)	−.27 (.13)	.41 (.14)	.21 (.10)	-
		*ρ* (*SD*_ρ_)	.51 (.16)	−.23 (.14)	−.31 (.14)	.49 (.19)	.24 (.10)	-
		CI	[.46, .56]	[ − .30, − .17]	[ − .38, − .24]	[.43, .56]	[.18, .30]	-
		CV	[.25, .77]	[ − .46, − .01]	[ − .55, − .08]	[.18, .80]	[.08, .41]	-

*Note.* CWB  =  counterproductive work behavior; *k*  =  number of independent samples; *N*  =  sample size; *r*  =  sample size-weighted mean uncorrected correlation; *SD_r_*  =  standard deviation of uncorrected correlation; *ρ*  =  mean corrected correlation (corrected for unreliability in predictor and criterion); *SD_ρ_*  =  standard deviation of corrected correlation; CI  =  95% confidence interval [lower value, upper value]; CV  =  90% credibility interval [lower value, upper value].

### Main Analyses

Prior to examining the full model, we first conducted analyses examining each mechanism in isolation as a point of comparison to our main model that included shared variance of all mechanisms. Results indicate that when examined singly, three out of the four proposed mechanisms mediated the relationship between leader mistreatment and CWB in the anticipated manner. Specifically, the indirect effect was significant for SERQ (indirect effect  =  .019, 95% CI [.013, .025]), state negative affect (indirect effect  =  .162, 95% CI [.149, .175]), and self-regulatory capacity impairment (indirect effect  =  .022, 95% CI [.014, .030]). However, interpersonal justice perceptions did not mediate the relationship between leader mistreatment and CWB (indirect effect  =  −.018, 95% CI [ − .038, .002])^3^.

**Figure 2.**
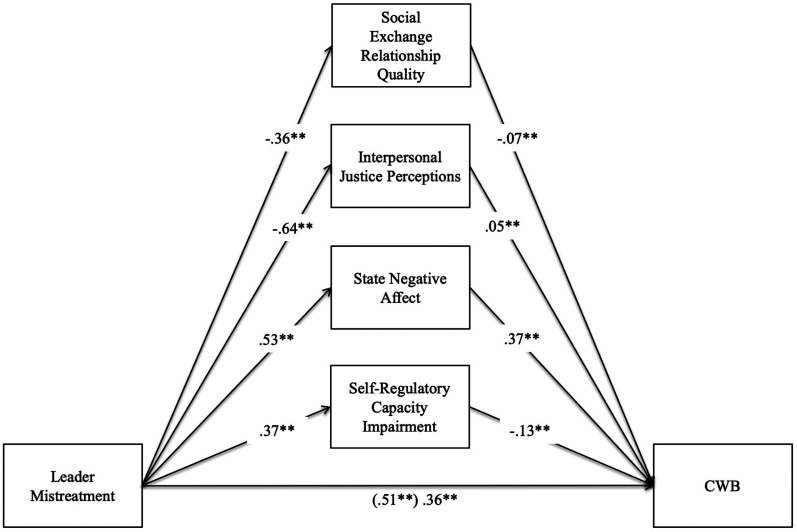
Structural equation modeling results for main analysis. *Note.* Standardized estimates. CWB  =  counterproductive work behavior. *p < .05, ** *p* < .01.

***Relative strength of the mediators.*** A slightly different picture emerges when the mediators are included in the model together. Figure 2 depicts the standardized path coefficients of our overall model, and the results of our simultaneous mediation tests are shown in Table 2. Consistent with *Hypothesis 1*, the direct effect of leader mistreatment on CWB is significant and positive (*β*  =  .51). Supporting *Hypothesis 2*, SERQ significantly mediated the relationship between leader mistreatment and CWB (indirect effect  =  .025, 95% CI [.015, .035]). Partially supporting *Hypothesis 3*, interpersonal justice perceptions significantly mediated the relationship between leader mistreatment and CWB, albeit in the opposite direction than our prediction (indirect effect  =  −.030, 95% CI [ − .051, − .009]). Supporting *Hypothesis 4*, state negative affect significantly mediated the relationship between leader mistreatment and CWB (indirect effect  =  .196, 95% CI [.178, .214]). Finally, partially supporting *Hypothesis 5*, impaired self-regulatory capacity significantly mediated the relationship between leader mistreatment and CWB, albeit in the opposite direction than our prediction (indirect effect  =  −.048, 95% CI [ − .059, − .038])^4^. However, when using the more conservative sample size of the smallest cell in the meta-analytic matrix (*N*  =  1074), as suggested by some researchers (e.g., Beus et al., 2015; Joseph et al., 2015), interpersonal justice perceptions no longer mediates the relationship between leader mistreatment and CWB (indirect effect  =  −.030, 95% CI [ − .070, .011]), in line with what was observed when it was examined alone as the mediator.

**Table 2.** Tests of Mediation for the Relationship Between Mistreatment to CWB.

Mediators	Leader mistreatment (X) → Mediators (M) → CWB (Y)	95% Bootstrapped Confidence Interval	
	Indirect effect	Lower	Upper
Social exchange relationship quality	0.025	0.015	0.035
Interpersonal justice perceptions	−0.030	−0.051	−0.009
State negative affect	0.196	0.178	0.214
Self-regulatory capacity impairment	−0.048	−0.059	−0.038

*Note.* Harmonic *N*  =  4124. CWB  =  counterproductive work behavior.

Finally, to answer *Research Question 1* regarding the mechanism that most strongly explains the relationship between leader mistreatment and employee CWBs, the mediated relationship via negative affect is significantly different from mediated relationships via SERQ (indirect effect difference  =  .171, 95% CI [.150, .192]), interpersonal justice perceptions (indirect effect difference  =  .226, 95% CI [.198, .253]), and impaired self-regulatory capacity (indirect effect difference  =  .244, 95% CI [.223, .265]), respectively. Overall, these results indicate that state negative affect was the strongest explanatory variable underlying the relationship between leader mistreatment and employee CWBs amongst the tested mechanisms.

Additionally, we conducted Relative Weight Analysis (RWA) to determine the relative importance of the predictors. As our predictors are correlated, we used the epsilon statistic to determine relative importance of each predictor, following recommendations by Tonidandel and LeBreton (2011) and Johnson (2000). The epsilon estimates, or relative weights, sum to the model *R*^2^ value and indicates each predictor’s respective, proportional, and direct contribution to *R*^2^ when combined with other predictors. Said another way, we can determine the *R*^2^ percentage that each predictor contributes by dividing the predictor’s relative weight by the model *R*^2^ value. This ease of interpretability is the reason why the epsilon statistic is preferred for computing relative importance (Johnson & LeBreton, 2004), so we use this statistic to assess the relative predictive validity of each predictor on CWB.

Overall, the mediators explain 27% of the variance in CWB. State negative affect emerged as the most important predictor of CWB, and it accounts for 40.3% of the total explained *R*^2^, followed by interpersonal justice perceptions (accounts for 8.8% of total explained *R*^2^), self-regulatory capacity impairment (accounts for 8.4% of total explained *R*^2^, and social exchange quality (accounts for 6.8% of total explained *R*^2^). Collectively, the results of RWA suggest that state negative affect is the most important mediator as it explains the most variance in CWB compared to the other mediators. These results are consistent with our structural equation modeling results in that state negative affect is the strongest mediator among all underlying mechanisms.

### Moderator Analyses

To answer *Research Question 2* regarding whether the mechanism that most strongly explains the relationship between leader mistreatment and employee CWBs generalizes across cultures varying in power distance, we ran two separate SEM models, one for high power distance and one for low power distance cultures, with available mediators (i.e., SERQ, state negative affect, and self-regulatory capacity impairment) included in the model simultaneously (see Table 3 for meta-analytic matrices, and Table 4 for indirect effects)^5^. In low power distance contexts, the mediated relationship via negative affect (indirect effect  =  .189, 95% CI [.169, .209]) is the strongest, and is significantly different from the mediated relationships via SERQ (indirect effect  =  .010, 95% CI [.000, .021]; indirect effect difference  =  .179, 95% CI [.157, .202]) and impaired self-regulatory capacity (indirect effect  =  −.032, 95% CI [ − .044, − .019]; indirect effect difference  =  .221, 95% CI [.198, .244]). In high power distance contexts, the mediated relationship via negative affect (indirect effect  =  .445, 95% CI [.398, .493]) is again the strongest, and is also significantly different from mediated relationships via SERQ (indirect effect  =  .110, 95% CI [.087, .134]; indirect effect difference  =  .335, 95% CI [.283, .388]) and impaired self-regulatory capacity (indirect effect  =  −.143, 95% CI [ − .170, − .116]; indirect effect difference  =  .588, 95% CI [.534, .643]). Thus, negative affect appears to be the most important explanatory variable underlying the leader mistreatment-CWB relationship across cultures varying in power distance^6^.

**Table 3.** Meta-Analytic Correlation Matrix for Low and High Power Distance Subgroups.

Variable			1	2	3	4	5
1.	Leader mistreatment	*k* (*N*)	-	8 (2573)	3 (1540)	4 (1105)	4 (1179)
		*r* (*SD_r_*)	-	− .29 (.12)	.49 (.11)	.34 (.07)	.30 (.08)
		*ρ* (*SD*_ρ_)	-	− .36 (.15)	.60 (.07)	.38 (.06)	.39 (.21)
		CI	-	[ − .47, − .25]	[.51, .69]	[.29, .46]	[.17, .60]
		CV	-	[ − .60, − .12]	[.49, .71]	[.28, .47]	[.04, .73]
2.	Social exchange relationship	*k* (*N*)	18 (6176)	-	3 (774)	6 (1713)	5 (1588)
	quality (SERQ)	*r* (*SD_r_*)	−.29 (.12)	-	− .09 (.10)	− .33 (.11)	−.17 (.04)
		*ρ* (*SD*_ρ_)	−.34 (.15)	-	− .10 (.16)	− .43 (.17)	−.21 (.03)
		CI	[ − .42, − .27]	-	[ − .30, .10]	[ − .58, − .28]	[ − .28, − .15]
		CV	[ − .60, − .09]	-	[ − .36, .16]	[ − .72, − .14]	[ − .27, − .16]
3.	State Negative affect	*k* (*N*)	12 (3103)	6 (908)	–	3 (501)	8 (1997)
		*r* (*SD_r_*)	.42 (.14)	−.29 (.13)	–	.43 (.02)	.43 (.15)
		*ρ* (*SD*_ρ_)	.49 (.15)	−.33 (.15)	–	.53 (.00)	.56 (.31)
		CI	[.40, .58]	[ − .48, − .19]	–	[.51, .55]	[.34, .78]
		CV	[.25, .74]	[ − .59, − .08]	–	[.53, .53]	[.05, 1.07]
4.	Self-regulatory capacity	*k* (*N*)	10 (3399)	24 (6240)	10 (3796)	–	3 (1285)
	impairment	*r* (*SD_r_*)	.35 (.09)	−.38 (.10)	.52 (.10)	–	.13 (.04)
		*ρ* (*SD*_ρ_)	.39 (.08)	−.46 (.08)	.61 (.12)	–	.14 (.00)
		CI	[.33, .46]	[ − .50, − .42]	[.53, .68]	–	[.08, .20]
		CV	[.26, .53]	[ − .59, − .33]	[.42, .80]	–	[.14, .14]
5.	CWB	*k* (*N*)	30 (11018)	10 (4089)	22 (5681)	10 (2855)	–
		*r* (*SD_r_*)	.42 (.14)	−.18 (.12)	.42 (.13)	.25 (.10)	–
		*ρ* (*SD*_ρ_)	.48 (.14)	−.23 (.16)	.50 (.14)	.29 (.10)	–
		CI	[.43, .54]	[ − .33, − .12]	[.43, .56]	[.22, .37]	–
		CV	[.25, .72]	[ − .49, .04]	[.26, .73]	[.14, .45]	–

*Note.* Low power distance subgroup correlations are reported below the diagonal; high power distance subgroup correlations are reported above the diagonal. CWB  =  counterproductive work behavior; *k*  =  number of independent samples; *N*  =  sample size; *r*  =  sample size-weighted mean uncorrected correlation; *SD_r_*  =  standard deviation of uncorrected correlation; *ρ*  =  mean corrected correlation (corrected for unreliability in predictor and criterion); *SD_ρ_*  =  standard deviation of corrected correlation; CI  =  95% confidence interval [lower value, upper value]; CV  =  90% credibility interval [lower value, upper value].

**Table 4.** Results of Moderator Analyses of Region on Relationships Between Leader Mistreatment and CWB as Mediated by Social Exchange Relationship Quality, State Negative Affect, and Self-Regulatory Capacity Impairment.

Mediators	Leader mistreatment (X) → Mediators (M) → CWB (Y)	95% Bootstrapped Confidence Interval	
	Indirect effect	Lower	Upper
Social exchange relationship quality			
Low power distance	0.010	0.000	0.021
High power distance	0.110	0.087	0.134
State negative affect			
Low power distance	0.189	0.169	0.209
High power distance	0.445	0.398	0.493
Self-regulatory capacity impairment			
Low power distance	−0.032	−0.044	−0.019
High power distance	−0.143	−0.170	−0.116

*Note.* Harmonic *N*  =  3160 for low power distance, harmonic *N*  =  1167 for high power distance. CWB  =  counterproductive work behavior.

To answer *Research Question 3* regarding whether the mediators have a weaker or stronger mediating effect across cultures varying in power distance, we compared the confidence intervals of the indirect effects of SERQ, negative affect, and impaired self-regulatory capacity in high and low power distance subgroups. Non-overlapping confidence intervals indicate significant differences between these two subgroups. Contrary to expectations, the mediating effect of SERQ is significantly *stronger* in high power distance cultures (indirect effect  =  .110, 95% CI [.087, .134]) than in low power distance cultures (indirect effect  =  .010, 95% CI [.000, .021]). Moreover, the mediating effect of negative affect was stronger in high power distance cultures (indirect effect  =  .445, 95% CI [.398, .493]) than in low power distance cultures (indirect effect  =  .189, 95% CI [.169, .209]), and the mediating effect of self-regulatory capacity impairment was stronger in high power distance cultures (indirect effect  =  −.143, 95% CI [ − .170, − .116]) than in low power distance cultures (indirect effect  =  −.032, 95% CI [ − .044, − .019]).

### Supplementary Analyses

***Serial mediation effects.*** Our results suggest that among the mediators that we examined, state negative affect most strongly accounts for the relationship between leader mistreatment and CWB. However, it is also possible that the other mediators influence CWB *via* state negative affect. Indeed, past research suggests a negative relationship between SERQ and negative affect (e.g., Conway et al., 2011; Conway & Briner, 2002), a negative relationship between interpersonal justice perceptions and negative affect (Barsky & Kaplan, 2007; Colquitt et al., 2013), and a positive relationship between impaired self-regulatory capacity and negative affect (Hagger et al., 2010). Thus, leader mistreatment might influence SERQ, interpersonal justice perceptions, and self-regulatory capacity, which in turn influence state negative affect, the most proximal predictor of CWB.

**Figure 3.**
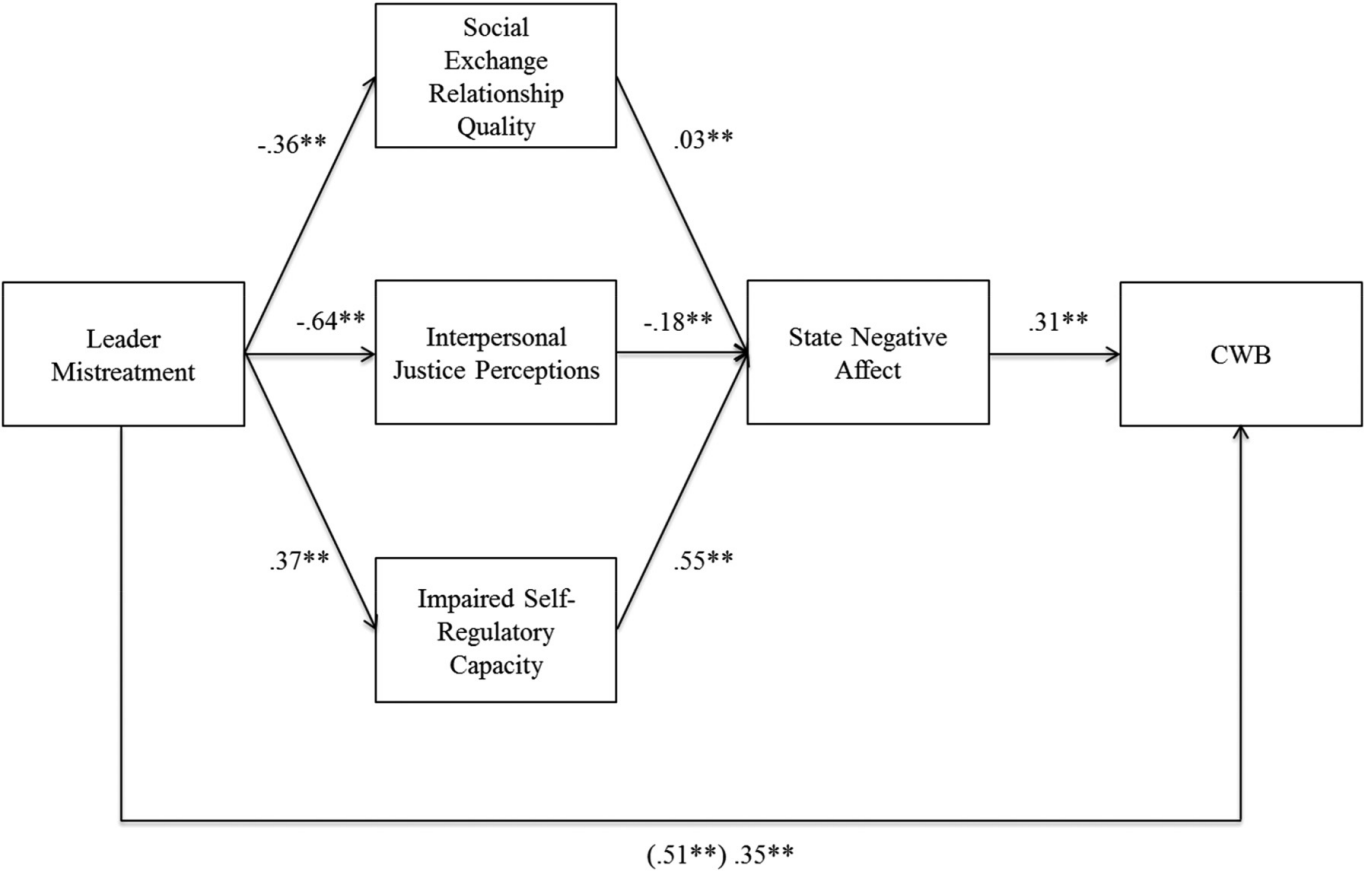
Structural equation modeling results for the serial mediation analysis. *Note.* Standardized estimates. CWB  =  counterproductive work behavior. *p < .05, ** *p* < .01.

We tested this alternative model (see Figure 3 and Table 5) and found that, indeed, the relationship between leader mistreatment and state negative affect is significantly mediated by SERQ (indirect effect  =  .020, 95% CI [.018, .023]), interpersonal justice perceptions (indirect effect  =  .067, 95% CI [.062, .071]), and impaired self-regulatory capacity (indirect effect  =  .209, 95% CI [.024, .214]). In turn, state negative affect significantly predicts CWB (*β*  =  .32, *p* < .001). The serial mediated relationships between leader mistreatment to CWB via first-order mediators (SERQ, interpersonal justice perceptions, and impaired self-regulatory capacity) and the second-order mediator (state negative affect) are significant (indirect effect  =  .006, 95% CI [.006, .007] for SERQ; indirect effect  =  .012, 95% CI [.011, .013] for interpersonal justice perceptions; indirect effect  =  −.066, 95% CI [ − .068, − .064] for impaired self-regulatory capacity). Moreover, the strengths of the three serial mediation paths are significantly different from one another: the path via interpersonal justice perceptions is the strongest positive effect, the path via impaired self-regulatory capacity is the strongest negative effect, and the path via SERQ is the smallest effect (indirect effect difference  =  −.005, 95% CI [ − .007, − .004] for SERQ vs. interpersonal justice; indirect effect difference  =  .072, 95% CI [.070, .075] for SERQ vs. impaired self-regulatory capacity; indirect effect difference  =  .078, 95% CI [.075, .080] for interpersonal justice vs. impaired self-regulatory capacity).

**Table 5.** Tests of Serial Mediation for the Relationship Between Mistreatment to CWB.

Mediators	Leader mistreatment (X) → First-Order Mediators (M1) → State Negative Affect (M2)	95% Bootstrapped Confidence Interval		Leader mistreatment (X) → First-Order Mediators (M1) → Second-Order Mediator (M2) → CWB (Y)	95% Bootstrapped Confidence Interval	
	Indirect effect	Lower	Upper	Indirect effect	Lower	Upper
Social exchange relationship quality	−0.012	−0.021	−0.003	−0.004	−0.007	−0.001
Interpersonal justice perceptions	0.112	0.095	0.129	0.034	0.028	0.040
Self-regulatory capacity impairment	0.205	0.187	0.223	0.063	0.055	−0.070

*Note.* Harmonic *N*  =  4124. CWB  =  counterproductive work behavior.

***Target-specific effects.*** Although in the current paper we do not make specific or differential predictions by the target of CWBs, consistent with prior research on the multi-foci perspective of CWBs (Chang & Lyons, 2012), we explore whether target-specific variables tend to be associated more strongly with target-specific CWBs. Specifically, we expect SERQ and interpersonal justice perception to predict target-specific CWBs more strongly than other-target CWBs (e.g., CWBs directed toward the organization) and serve as more important mediators of the relationship between leader mistreatment and supervisor-targeted CWBs. In contrast, state negative affect and self-regulatory capacity impairment are not necessarily tied to specific individuals. Thus, we expect them to predict CWBs directed toward various targets equally well and mediate the relationship between leader mistreatment and CWBs directed to different targets similarly. More generally, taking a multi-foci perspective helps us to better understand whether and how interventions could more effectively reduce different types of CWBs by focusing on target-specific or non-specific mechanisms (Hershcovis & Barling, 2010).

To test whether target-specific constructs better mediate the relationship between leader mistreatment and target-specific CWBs (i.e., directed at the supervisor vs. the organization)^7^, we recoded all available studies to separate the targets, which yields a subset of samples (*k*  =  156) with target-specific constructs. We conducted meta-analyses using these target-specific correlations, sample sizes, and reliabilities, using the same procedures as the main analysis, and constructed a meta-analytic correlation matrix. For constructs in which target is not relevant (e.g., negative affect) or did not vary in our data (e.g., mistreatment), meta-analytic correlations from the main analysis were used. As expected, an examination of the bivariate relationships indicate that target-specific mediators correlate more strongly with target-specific CWBs, such that SERQ-S has a stronger correlation with CWB-S (*ρ*  =  −.30, *k*  =  3, 95% CI [ − .565, − .039]) than with CWB-O (*ρ*  =  −.13, *k*  =  6, 95% CI [ − .185, − .078]), SERQ-O has a stronger correlation with CWB-O (*ρ*  =  −.27, *k*  =  11, 95% CI [ − .330, − .205]) than with CWB-S (*ρ*  =  −.17, *k*  =  6, 95% CI [ − .248, − .099]), and interpersonal justice perceptions has a stronger correlation with CWB-S (*ρ*  =  −.35, *k*  =  10, 95% CI [ − .458, − .234]) than with CWB-O (*ρ*  =  −.17, *k*  =  9, 95% CI [ − .212, − .127]). Moreover, the non-target-specific mediators correlated with both CWB-S and CWB-O in a similar magnitude: state negative affect is correlated with CWB-S (*ρ*  =  .55, *k*  =  8, 95% CI [.476, .631]) and CWB-O (*ρ*  =  .51, *k*  =  12, 95% CI [.451, .577]), and self-regulatory impairment is correlated with CWB-S (*ρ*  =  .22, *k*  =  6, 95% CI [.113, .324]) and CWB-O (*ρ*  =  .26, *k*  =  3, 95% CI [.057, .453]).

**Figure 4.**
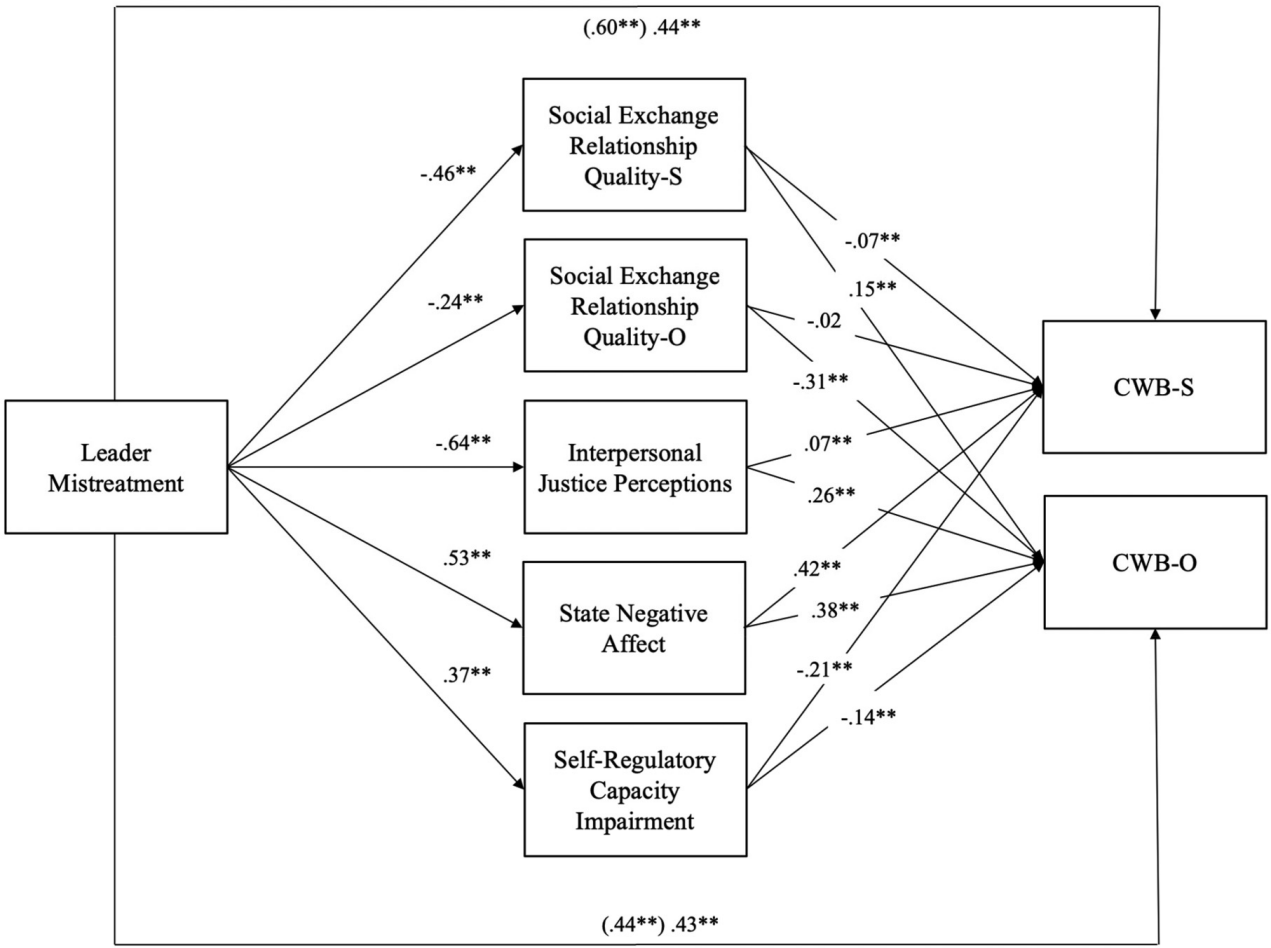
Structural equation modeling results for target-specific analysis. *Note.* Standardized estimates. S  =  Supervisor-directed, O  =  Organization-directed, CWB  =  counterproductive work behavior. *p < .05, ** *p* < .01.

We then ran an SEM model with leader mistreatment regressed on both CWB-S and CWB-O with all mediators (i.e., SERQ-S, SERQ-O, interpersonal justice perceptions, state negative affect, and self-regulatory capacity impairment) included in the model simultaneously^8^. Figure 4 depicts the SEM results of target-specific analyses. For target-specific mediators, consistent with what we expected, SERQ-S significantly mediated the relationship between leader mistreatment and CWB-S (indirect effect  =  .031, 95% CI [.017, .045]), whereas SERQ-O did not mediate (indirect effect  =  .005, 95% CI [ − .002, .012]). However, contrary to our expectations, interpersonal justice perceptions mediated the relationship from leader mistreatment to CWB-O (indirect effect  =  −.173, 95% CI [ − .199, − .147]) more strongly compared to CWB-S (indirect effect  =  −.049, 95% CI [ − .072, − .026]; indirect effect difference  =  .124, 95% CI [.106, .143]).

An examination of non-target-specific mediators revealed that, state negative affect significantly mediated the relationship between leader mistreatment and CWB-S (indirect effect  =  .231, 95% CI [.209, .253]) and CWB-O (indirect effect  =  .212, 95% CI [.189, .234]); unexpectedly, the strength of the mediated effect was stronger for CWB-S compared to CWB-O (indirect effect difference  =  .019, 95% CI  =  [.006, .033]). Similarly, self-regulatory capacity impairment significantly mediated the relationship between leader mistreatment and CWB-S (indirect effect  =  −.079, 95% CI [ − .092, − .066]) and CWB-O (indirect effect  =  −.055, 95% CI [ − .068, − .042]); again, unexpectedly, the strength of the mediated effect was stronger for CWB-S compared to CWB-O (indirect effect difference  =  −.024, 95% CI  =  [ − .033, − .015]).

***Time-lagged effects.*** Although we are primarily interested in how leader mistreatment results in employee CWBs in our meta-analysis, the reverse causal ordering of CWBs predicting leader mistreatment is not only possible, but also supported by both theory and research (e.g., Lian et al., 2014a; Simon et al., 2015). Given that studies included in our meta-analysis are mostly cross-sectional in nature, and we coded relationships based on the same, shortest, and earliest time point, our results cannot strongly speak to the directionality of our hypothesized effect (Maxwell & Cole, 2007; Ployhart & Vandenberg, 2010). Nevertheless, we identified 62 articles (79 studies) that measured leader mistreatment and CWBs at separate occasions. Within these studies, we identified 17 articles (22 studies) that measured CWBs with at least a one-day time lag after leader mistreatment; the average lag was 3 months. Meta-analytic results indicate that the lagged relationship between leader mistreatment and CWBs (*ρ*  =  .51, *k*  =  22, 95% CI [.440, .579]) is exactly the same as our overall estimate (*ρ*  =  .51, *k*  =  50, 95% CI [.464, .556]). Unfortunately, because very few studies employed a panel design (i.e., repeated measurement of the same variable across time), we could not examine reciprocal effects.

## Discussion

The purpose of this meta-analytic review was to understand *why* leader mistreatment relates to CWB. Specifically, within the past two decades, process models drawing on the social exchange perspective (Mitchell & Ambrose, 2007), the justice perspective (Tepper, 2000), the stressor-emotion perspective (Spector & Fox, 2005), and the self-regulatory capacity perspective (Thau & Mitchell, 2010), have all been featured prominently in high-quality publications in explaining the relationship between leader mistreatment and CWB. However, as researchers generally do not directly compare the explanatory power of these theories (Davis, 2010), the leader mistreatment literature has suffered from theory proliferation (Harter & Schmidt, 2008). That is, when testing theories independently, researchers tend to only seek confirmatory evidence that is consistent with a preferred theoretical account, without questioning whether other plausible theoretical accounts also apply (Greenwald et al., 1986). Indeed, empirical studies in this literature have typically focused only on a given mechanism, resulting in confirmations of different theories and mediating mechanisms that may explain the same phenomenon and yet are tested independently of one another.

In order to move beyond this theoretical stalemate, we empirically pitted these theoretically derived mediating mechanisms against one another in our meta-analysis. We meta-analyzed 168 articles (214 studies) and used MASEM to test the strength of four key mediators derived from these theoretical perspectives. By testing mediators independently as well as together in a model, we underscore the value of using a meta-analytic framework to compare the effect of various mechanisms in the leader mistreatment-CWB relationship. When we tested the mechanisms independently and did not account for their shared variance, as most individual studies do, we show that all mechanisms except for interpersonal justice perceptions significantly mediate the relationship. Yet, when the mediators were placed in the model together, the unique effect of each mediator becomes clearer, such that only SERQ and state negative affect significantly mediated the relationship in the theoretically expected manner—and SERQ only weakly so—leaving state negative affect as the “champion” (Leavitt et al., 2010, p. 644). Further, this appears to be the case across cultures varying in power distance.

### Theoretical Implications

***Relative strength of the mediators.*** Our findings contribute to a better understanding of the workplace mistreatment literature by reducing the number of probable mechanisms in explaining the leader mistreatment-CWB relationship. When the four mechanisms are analyzed in isolation, all but one (interpersonal justice) was found to be a significant mediator of the relationship between leader mistreatment and CWB. Whereas this is generally consistent with past research, by examining the mediators together, we also reveal that the mediators differ in strength. That is, mediators drawn from social exchange, justice, and self-regulatory capacity perspectives did not provide strong and unique explanations for the link between leader mistreatment and CWB.

Notably, despite theoretical popularity and intuitive appeal, we found that interpersonal justice perception is unlikely to be a meaningful mediator of leader mistreatment and CWB. Although it was a significant mediator in one model in which other mediators were included, when using a more conservative sample size and when it was examined in isolation, it was not a significant mediator. Thus, on the whole, the evidence for interpersonal justice perceptions as an important mediator of leader mistreatment and CWB is quite weak. Our results also correspond to prior meta-analyses (Zhang et al., 2019) in which organizational justice was not a consistently significant mediator of abusive supervision and CWB. Note that consistent with past research, we found significant bivariate relationships between leader mistreatment and interpersonal justice perceptions (*ρ*  =  −.64) and between interpersonal justice perceptions and CWBs (*ρ*  =  −.31). However, it is important to formally test mediation because leader mistreatment, which is strongly related to both justice (*ρ*  =  −.64) and CWB (*ρ*  =  .51), can act as a confound or a third variable when the bivariate relationship between justice and CWB is examined in isolation. Supporting this idea, the relationship between justice and CWB decreases to .03 and is not significant in our mediation model. Thus, we argue that we obtained a more accurate estimate of the indirect effect of mistreatment on CWB via interpersonal justice perception than what might be presumed based on existing literature. Theoretically, our findings suggest that interpersonal justice perceptions may not be a viable explanation for the relationship between leader mistreatment and CWB. This is noteworthy because, given the ethical implications of leader mistreatment, the deontic model of justice is highly appealing as a theoretical explanation for why employees engage in CWBs in response to leader mistreatment.

Moreover, although self-regulatory capacity impairment was a significant mediator of the relationship between leader mistreatment and CWB, when controlling for other mediators, greater self-regulatory capacity impairment led to *less* CWB. The indirect effect via self-regulatory capacity impairment was in the expected direction when this mediator was analyzed on its own. This suggests that the other mediators account for the portion of variance in self-regulatory capacity impairment that is positively related to CWB and its unique variance is negatively related to CWB. The shared portion of variance might represent the *desire* to engage in CWBs. Self-regulatory capacity impairment leads individuals to engage in behaviors that they already desire to perform (Baumeister & Heatherton, 1996) and negative emotions or a damaged exchange relationship may be sources for desires to engage in CWB. Thus, the positive relationship between self-regulatory capacity impairment and CWBs may be explained by underlying desires to engage in CWBs. On the other hand, self-regulatory capacity impairment might diminish a person’s *ability* to engage in CWBs, which is a process that may not be shared with other constructs. For example, there is some evidence that engaging in CWB can sometimes require *more* effort than refraining from CWBs, and thus individuals may engage in *less* CWB when their self-regulatory capacity is impaired (Yam et al., 2014). Although speculative, this suggests that future research on leader mistreatment and CWB that draws on self-regulatory capacity framework may need to consider these different aspects of self-regulatory capacity.

Our focus on a broad range of commonly invoked theoretical mechanisms (i.e., a social exchange perspective, a justice perceptive, an impaired self-capacity resource perspective, and a negative affect perspective) moves beyond prior investigations by being more comprehensive. In particular, we found that negative affect is the strongest mediating mechanism in explaining why employee CWBs occur in response to leader mistreatment. Our supplementary analyses also revealed that SERQ, interpersonal justice, and self-regulatory capacity impairment influence CWB via negative affect, which suggests that although the other mediators may play important roles in explaining the relationship between leader mistreatment and CWB, they might do so via negative affect. Thus, our research reveal that negative affect is a key mechanism explaining the relationship between leader mistreatment and CWB, which is something that has not been identified by prior meta-analyses (i.e., Zhang et al., 2019).

Moreover, although prior meta-analyses (i.e., Zhang et al., 2019) has examined cultural moderators (i.e., masculinity/femininity) in abusive supervision-CWB relationship in that the relationship is stronger in masculine cultures, our meta-analysis took a different angle and has shown that regardless of cultural differences in power distance, negative affect emerged as the strongest mediator in comparison to the other mediators. Given these important findings, we recommend that CWB researchers adopt an affective theoretical lens to understand why employees commit deviance when they are mistreated. Accordingly, given that we have little current knowledge about *how* negative affect functions as a mechanism, our research opens potentially fruitful theoretical and empirical lines of inquiry in investigating the link between mistreatment and CWB. Specifically, we identify the need for a better articulation of affective process theories and for studying how affect functions as a mechanism for spurring CWB, perhaps through identifying and investigating boundary effects (workplace characteristics) that weaken the effect.

In sum, continuing to draw on social exchange, justice, or self-regulatory capacity perspectives to explain why leader mistreatment leads to employee CWB might not be productive. The basic tenets of these perspectives may be useful for guiding research on leader mistreatment and CWB, yet researchers should consider ways in which these perspectives may be limited. That is, to develop a parsimonious and strong theory that explains why leader mistreatment leads to employee CWB, it may be beneficial to exclude, modify, or integrate these theoretical perspectives. Moreover, we show that the direct relationship between mistreatment and CWB remains significant even when we test all four mediators simultaneously. This significant unaccounted-for variance suggests that there may be other possible mechanisms in the mistreatment-CWB relationship. We discuss other possible theoretical explanations in the study limitations and future directions section below.

***Implications for emotions research.*** Despite being the least frequently studied mechanism among the four commonly invoked theoretical perspectives, state negative affect emerged as the strongest explanatory mechanism. This discovery calls attention to the emotions literature by highlighting the need for more nuanced theoretical perspectives, particularly for state negative affect, in explaining why employees engage in CWBs in reaction to leader mistreatment. That is, a commonly invoked model for the effects of emotions at work is the stressor-emotion model of CWBs (Spector & Fox, 2005), which suggests that experiencing negative emotions lead to CWB. However, this model does not provide a compelling explanation for why or how negative emotions lead to CWB. We expanded on the stressor-emotion model and argued that employees might use CWBs to cope with their emotions (e.g., Krischer et al., 2010), because they might believe that CWBs can improve their affect. Thus, to provide a strong account for why leader mistreatment leads to employee CWBs, integration of theories from organizational and emotion literatures may be needed. Moreover, other explanations for the role of negative affect may also be relevant. Such alternatives and possible future avenues are discussed in the limitations and future directions section.

***Implications for cross-cultural research.*** We contribute to cross-cultural organizational research by demonstrating stability and variation in the effects we found as a function of cultural differences in power distance. Despite growing interests in leader mistreatment among researchers across the world, cross-cultural studies of leader mistreatment are rare (Tepper et al., 2017), possibly because conducting cross-cultural studies can be highly resource-intensive. On the other hand, meta-analysis is a convenient and cost-effective method to examine the effects of culture. Given that studies on leader mistreatment have been conducted in multiple countries (Martinko et al., 2013), we imputed the power distance score of the country from which the sample was drawn to examine how cultural values may moderate relationships within our model.

Our results show that, most notably, relative to SERQ and self-regulatory capacity impairment, state negative affect was the strongest mediator across cultures varying in power distance. Thus, whereas cross-cultural researchers may focus on differences across cultures, our findings highlight a potentially universal mechanism for why leader mistreatment leads to employee CWBs. That is, the non-shared predictive power of these mechanisms may be largely invariant across cultures.

However, comparing each mediator across cultures revealed unexpected findings. First, although we expected mistreatment to be less likely to damage SERQ perceptions for individuals in high (vs. low) power distance cultures, mistreatment-SERQ relationship was similar in magnitude across low (*β*  =  −.34) and high (*β*  =  −.36) power distance cultures, indicating that mistreatment is equally damaging to SERQ perceptions across varying cultural contexts. We instead found that power distance moderates the downstream SERQ-CWB relationship. That is, SERQ was more strongly and negatively related to CWB in high power distance cultures (*β*  =  −.27) compared to low power distance cultures (*β*  =  −.03). This might be because, in high power distance cultures, the value placed on social hierarchy and acceptance of the leader’s power may also lead to employees’ increased focus on maintaining a stable exchange relationship with their leaders. This might result in stronger reactions when the stability of this relationship is threatened. In contrast, employees in low power distance cultures may place less value on their exchange relationship with their leaders and may therefore react less strongly when their relationship with their leader is threatened. Thus, employees in high power distance cultures might react more strongly and more negatively (i.e., by performing CWBs) compared with employees in low power distance cultures.

Moreover, contrary to our expectations, negative affect was a stronger mediator in high power distance cultures than low power distance cultures. Specifically, power distance moderated the negative affect-CWB relationship, such that individuals in high power distance cultures who experienced negative affect were more likely to engage in CWB (*β*  =  .65) compared to individuals in low power distance cultures (*β*  =  .38). We speculate that if individuals tend to suppress and experience less negative affect in high power distance cultures compared to low power distance cultures (Matsumoto et al., 2008), negative affect may be more diagnostic of individuals’ behavior in high power distance cultures. That is, for individuals in high power distance cultures, experiencing (and self-reporting) a high level of negative affect might be unusual and may suggest that they are particularly in need of coping with their emotions, whereas a high level of negative affect may not be as unusual for individuals in low power distance cultures.

Finally, contrary to our expectations, self-regulatory capacity impairment was a stronger mediator in high power distance cultures than low power distance cultures. Specifically, we found that leader mistreatment affects employees’ self-regulatory capacity impairment equally in low (*β*  =  .39) and high (*β*  =  .38) power distance cultures. However, individuals in high power distance cultures engage in *less* CWB as they feel *more* exhausted (*β*  =  −.33) whereas individuals in low power distance cultures engage in more CWB as they feel more exhausted (*β*  =  .08). We observed these findings when controlling for SERQ and negative affect. SERQ and negative affect might provide motives for individuals to engage in CWBs *when* they are depleted. Thus, holding constant SERQ and negative affect, when self-regulatory capacity is impaired as a result of leader mistreatment, individuals in high power distance cultures might be less able to engage in CWBs than individuals in low power distance cultures. This is because engaging in behaviors that go against one’s leader or organization might be challenging in a culture that values and accepts social hierarchy and may require a high level of self-regulatory capacity. On the other hand, individuals in low power distance cultures might lack such a barrier against engaging in CWBs towards one’s leader or organization (due to lower acceptance of social hierarchy), which may increase the likelihood that they will engage in CWBs when they are depleted.

In sum, our analyses of power distance as a national culture moderator highlight the need for a nuanced approach to examining the ways in which cultural values play a role in explaining the relationship between leader mistreatment and CWB. In particular, given the moderation happens mostly at the second stage (i.e., the mediator to CWB link) rather than the first stage (i.e., leader mistreatment to the mediators), it suggests people in cultures varying in power distance may interpret leader mistreatment similarly, but it is their reactions to those interpretations that drive the difference in CWB across cultural contexts. Moreover, given the importance of negative affect as a mechanism across cultures varying in power distance, drawing on theories and findings from cross-cultural research that examines emotional experience and expression may be a productive path toward a richer understanding of leader mistreatment and CWB.

***Implications for applying a multi-foci perspective.*** We contribute to research on multi-foci perspective by revealing instances in which applying this perspective to understand the link between leader mistreatment and CWB is beneficial. That is, consistent with the multi-foci perspective, SERQ with the supervisor was a significant mediator of the relationship between leader mistreatment and CWB directed at the supervisor, whereas SERQ with the organization was not. This reveals the importance of drawing on social exchange perspective in a nuanced way; researchers might commonly treat SERQ with the supervisor and organization interchangeably, yet we demonstrate that aggregating these constructs may result in misleading conclusions. Thus, at least for SERQ, multi-foci perspective may be an important theoretical perspective to integrate when examining why leader mistreatment leads to CWBs.

We expected that mediators that are not focused on specific targets (i.e., negative affect and self-regulatory capacity impairment) may not have differential effects based on the target of CWB. However, the indirect effect of mistreatment via negative affect was stronger for CWB-S than CWB-O. Although one might speculate that negative affect relating to one’s supervisor (e.g., hostile emotions toward the supervisor) might be more predictive of CWB-S than CWB-O, we in fact included studies that measured negative affect in various ways for this analysis (i.e., negative affect without reference to events or persons as well as specific emotions toward individuals at work). Thus, this finding may not be an artifact due to differences in measurement of negative affect. It is possible that employees target their supervisors when experiencing negative affect because they are more narrowly focused on their immediate social interactions. Research has shown that on average, negative affect tends to narrow one’s attention (e.g., Friedman and Förster, 2010; Schwarz and Clore, 2003). As for self-regulatory capacity impairment, it was more strongly negatively related to CWB-S than CWB-O. As we argued above regarding the role of self-regulatory capacity in the effort required to engage in CWBs, it is possible that CWB-S requires more effort than CWB-O. For example, acting rudely toward one’s supervisor may require employees to publicly violate clear social and organizational norms against such behaviors. On the other hand, CWB-Os, such as time theft and neglect might be more covert, and thus less effortful to do.

In sum, although we did not expect many of these differences based on target-specific perceptions and behaviors, our results highlight the contribution of the multi-foci perspective in the understanding of the link between leader mistreatment and employee CWB. As such, a theory of leader mistreatment and CWB should integrate ideas from the multi-foci perspective.

**Practical Implications**

Given that our results identified negative affect as the most important mechanism by which interpersonal leader mistreatment results in CWB, organizations could curtail the occurrence of CWB by providing training and interventions targeted at reducing negative affect when aversive events, such as leader mistreatment, occur. In addition to working to reduce interpersonal mistreatment at work, when attempting to help employees who are mistreated by others, interventions such as emotion regulation training or mindfulness training (e.g., Mindfulness-Based Cognitive Therapy) can provide methods to manage the emotional reactions caused by mistreatment. Mindfulness training would help employees to focus on the present moment, which would not only increase their awareness of how external workplace events affect them internally, but also minimize particular feelings, positive or negative, that are associated with these events (Jamieson & Tuckey, 2017). Thus, mindfulness training may help to alleviate high state negative affect in employees as a reaction to leader mistreatment, thereby decreasing the likelihood that employees would engage in CWBs.

In addition to reducing the impact of leader mistreatment on negative affect, interventions can aim to prevent employees who are experiencing negative emotions from engaging in CWBs, and instead channel their negative emotions toward less destructive actions. For instance, anger can motivate individuals to engage in less destructive methods of addressing leader mistreatment (e.g., petition against the leader) if they feel that they can enact change or if they strongly value ethical conduct (Mitchell et al., 2015; Priesemuth & Schminke, 2019; Turner, 2007). Thus, organizations can enhance employees’ sense that they can positively address leader mistreatment (e.g., by providing conflict resolution training) or increase their perceived importance of ethical conduct (e.g., by modeling just and ethical behaviors) to increase the likelihood that employees will engage in less destructive actions in response to leader mistreatment.

Finally, our findings suggest that organizations that work across different national cultures may need to consider a range of cultural factors when attempting to understand and reduce CWBs that stem from leader mistreatment. That is, whereas Zhang et al. (2019) found that leader mistreatment more strongly increases CWBs in masculine cultures than in feminine cultures, our results indicate that negative affect is an important mechanism underlying this relationship, particularly in high power distance cultures relative to low power distance cultures. This means that organizations that are concerned about CWBs may need to pay special attention to leader mistreatment occurring in masculine cultures, but may also need to consider the influence of power distance to prevent leader mistreatment from subsequently increasing employee CWBs.

Limitations and Future Directions

Despite the strength of meta-analytically synthesizing the literature and testing four disparate theories in one model, the current study has several limitations. The first limitation is that our meta-analysis is based on correlational studies that use, for the most part, cross-sectional designs. As such, we cannot draw causal inferences (Bergh et al., 2014). Only 45 out of the 214 studies (∼21%) measured relationships between variables separated by time. Although we provided evidence that the meta-analytic correlation between mistreatment and CWB computed from time-lagged studies were not different from cross-sectional studies, study designs may have nevertheless affected the indirect relationships between mistreatment and CWB. In particular, in most studies, interpersonal justice perceptions were measured at the same time as mistreatment, CWB, or both; we cannot rule out the possibility that this may have affected our findings regarding interpersonal justice as a mediator. To ensure that methodology better matches theory, researchers could invest in study designs that allow stronger causal claims, such as time-lagged studies and experiments.

The second limitation is that, even though we tested four theoretically-driven mediators, we were unable to test other possible mechanisms for the relationship between leader mistreatment and CWB. Indeed, after including all four mediators, there is still a substantial residual relationship between leader mistreatment and employee CWBs. Future studies will be informative if they examine other plausible explanations that were not examined in our investigation. For example, drawing on social learning theory, mistreated employees might engage in CWBs because they learn and emulate such mistreatment. Indeed, Lian et al. (2012b) proposed and found that employees who are exposed to leader mistreatment believe that such behaviors are rewarded, which is an important antecedent of social learning (Bandura, 1965). For the purposes of this meta-analysis, we chose not to investigate this and other mechanisms, first, due to the lack of consistency in operationalization in the limited primary studies (which we determined through a cursory review of the literature) and second, because we wanted to provide a strong test of commonly invoked theories in the literature.

The third limitation of this study is that although we identified negative affect as a potential mechanism explaining the relationship between leader mistreatment and CWB, the stressor-emotion perspective is theoretically ambiguous. We drew on literature on coping to theorize that CWB is a way to cope with negative emotions. However, other theoretical perspectives may have identified negative affect as a mediator but may have provided a different explanation. For example, employees who experience negative affect as a result of mistreatment may use their unpleasant feelings to justify their subsequent CWB as a reasonable response to the mistreatment. That is, employees might morally disengage from their actions (Fida et al., 2015). Moral disengagement is a process in which actors of deviant behavior use rationalizations to remove negative aspects of that behavior that would normally deter them from engaging in it (Fida et al., 2015). Because our results suggest that state negative affect is an important and unique mechanism that explains the relationship between leader mistreatment and CWB, more work is needed in this area to provide a more nuanced understanding of this mechanism.

As a fourth limitation, we also acknowledge and suggest that leader mistreatment may have indirect ramifications on other employee performance outcomes besides CWB via the four identified mediators. Many researchers have found medium-to-large effects of leader mistreatment on SERQ, interpersonal justice, self-regulatory capacity impairment, and negative affect (e.g., Mackey et al., 2015; Tepper, 2000; Xu et al., 2012). A previous meta-analysis by Mackey et al. (2015) looking at the effects of abusive supervision on employee outcomes found strong negative relationships between abusive supervision and two SERQ indicators, LMX (*ρ*  =  −.54, *k*  =  11, 95% CI [ − .70, − .39]) and POS (*ρ*  =  −.40, *k*  =  7, 95% CI [ − .55, − .25]), and between abusive supervision and supervisor interactional justice (*ρ*  =  −.39, *k*  =  5, 95% CI [ − .64, − .15]), as well as positive relationships between abusive supervision and emotional exhaustion (i.e., self-regulatory capacity impairment; *ρ*  =  .36, *k*  =  15, 95% CI [.21, .51]), and between abusive supervision and negative affect (*ρ*  =  .37, *k*  =  27, 95% CI [.19, .56]). Given these findings, all four mediators may act as plausible mechanisms for relationships between leader mistreatment and other employee performance outcomes, such as organizational citizenship behavior (OCB)—a discretionary behavior whereby individuals engage in actions not formally recognized by any reward system but that promote optimal organizational functioning (Organ, 1988). Many studies have found leader mistreatment to be a robust predictor of OCB, including the same meta-analysis by Mackey et al. (2015). Further, researchers have also found links between all four mediators—SERQ, interactional justice, negative affect, and self-regulatory capacity impairment—and OCB (e.g., Cropanzano et al., 2003; Geiger et al., 2007; Karriker & Williams, 2009; Xu et al., 2012). As such, leader mistreatment could potentially affect OCB indirectly through these four mediators, and more strongly through some mediators over others, as our research has found with CWB. Thus, we would recommend that future meta-analytic research test these four mediators as mechanisms for relationships between leader mistreatment and other employee outcomes, such as OCB.

The fifth limitation of this study is, although we recommend that organizations spanning multiple countries may need to consider many cultural factors to understand and reduce CWBs that stem from leader mistreatment, our research only explores the moderating effect of one of these cultural factors, power distance, on the relationship between leader mistreatment and CWBs in detail. We note that we also investigate the moderating effects of individualism-collectivism in the current paper; however, as our findings were largely similar to when power distance is the moderator (i.e., negative affect remained the strongest mechanism across individualistic and collectivist countries), we opted to save space and report these findings in a footnote. In addition, although our research was somewhat hampered from investigating the moderating effect of other cultural factors by a lack of studies, our hope is that this trend will reverse for future research on leader mistreatment and employee CWB. The moderating effect of culture on the indirect link between mistreatment and CWB is important to further elucidate, as our investigation of one cultural dimension may offer a limited view into the influence of culture on this relationship. For example, a previous meta-analysis looking at the effects of justice on employee outcomes across different countries found that the strength of these relationships depended on various cultural factors, such as power distance, masculinity-femininity, individualism-collectivism, and uncertainty avoidance (Shao et al., 2013). To that end, to provide a more comprehensive view on the effect of culture on mediators of the indirect leader mistreatment-employee CWB relationship, we urge future research to consider investigating other possible cultural factors that have not been presented in this research, such as masculinity-femininity and uncertainty avoidance (i.e., the extent to which individuals feel comfortable with ambiguous situations; Hofstede, 2011).

Finally, we examined each construct at a broad level, without differentiating between specific operationalizations (e.g., abusive supervision, supervisor incivility, supervisor undermining) in order to test the feasibility of the different theories. It can be argued that there might be meaningful differences between operationalizations, thus combining scales under a broad construct may not capture the nuances. To address this concern, we conducted supplementary analyses with only the most frequently occurring measures in our data, and the results and conclusion remained the same. Specifically, when leader mistreatment is solely operationalized with abusive supervision (*k*  =  76), it is positively and strongly correlated with employee CWBs (*ρ*  =  .51, *k*  =  50, 95% CI [.47, .55]) and its direct effect on CWB is positive and significant when the mediators are included in the model (*β*  =  .38, *p* < .001). Moreover, SERQ (indirect effect  =  .016, 95% CI [.005, .028]), interpersonal justice (indirect effect  =  −.026, 95% CI [−.048, − .005]), state negative affect (indirect effect  =  .192, 95% CI [.174, .209]), and impaired self-regulatory capacity (indirect effect  =  −.049, 95% CI [ − .061, − .038]) all significantly mediate the relationship between abusive supervision and CWB.

Similarly, when SERQ is solely operationalized with affective commitment (*k*  =  62) in a model with all the mediators included, the direct effect of leader mistreatment on CWB is significant and positive (*β*  =  .38, *p* < .001), and affective commitment (indirect effect  =  .021, 95% CI [.024, .040]), interpersonal justice (indirect effect  =  −.072, 95% CI [ − .065, − .022]), state negative affect (indirect effect  =  .172, 95% CI [.178, .216]), and impaired self-regulatory capacity (indirect effect  =  −.071, 95% CI [ − .068, − .045]) all significantly mediate the relationship between leader mistreatment and CWB. Moreover, negative affect still emerged as the strongest mediator in these models, further indicating that it is unlikely for our findings to have been driven by specific operationalizations of these broad constructs.

In fact, we argue that by using broad constructs rather than specific measures, our meta-analysis contributes to the parsimony of science. That is, advantages of narrow constructs (e.g., precision and clarity) must be balanced with usefulness and robustness of findings using broad constructs, and the decision to use broad constructs in meta-analyses must be informed by theory and empirical evidence (Viswesvaran & Ones, 1995). If two measures have similar definitions, have observed substantial correlations with each other, and predict other constructs similarly, then they may be empirically indistinguishable and should be considered as different operationalizations of the same construct (Le et al., 2010). For instance, different measures of leader mistreatment predict other constructs similarly (Hershcovis, 2011), various measures of SERQ (that we included in our analyses) correspond well to the construct definition of SERQ (Colquitt et al., 2014), and various forms of CWBs are described by a higher-order general factor (Marcus et al., 2013). Because our goal was to evaluate the different theoretical perspectives explaining the relationship between leader mistreatment and CWBs, it was important to focus on broad constructs. In future investigations, however, it would be informative to formally model these hierarchical structures such that broad constructs are modeled as higher-order factors of narrow constructs, using intercorrelations between different operationalizations (Viswesvaran & Ones, 1995)^9^.

## Conclusion

Taken together, the present meta-analytic review provides a theoretical integration by comparing the non-shared predictive power of four proposed mechanisms––social exchange relationship quality, interpersonal justice perceptions, state negative affect, and impaired self-regulatory capacity––in explaining the leader mistreatment and CWB relationship. This relationship is of critical interest as it underscores how harmful behaviors in the workplace spread from leaders to followers. When examined simultaneously, negative affect emerged as the strongest explanatory mechanism, and remained so in cultures varying in power distance. As such, our results highlight the unique variance of negative affect that is not shared with the other mediators in explaining the leader mistreatment-CWB relationship. These results not only provide an integration of the fragmented theoretical landscape which is rife with theories that are overlapping with one another, but also pave a way for researchers who are seeking to test novel mechanisms of the leader mistreatment-CWB relationship––as they may wish to engage in “strong inference” testing (i.e., Leavitt et al., 2010, p. 644) by pitting negative affect with the other mechanisms to gauge for the unique variance that they explain.

## Notes

1. We focused on correlational studies with minimal time lag because they were the most common and thus ensured two things: 1) that our meta-analysis is representative of studies that currently exist in the literature and can speak to current research; and 2) that we could test our full model, the purpose of our meta-analysis in the first place. Given that our research focus is on comparing competing mechanisms, our priority for this meta-analysis was in making sure that relationships in our model were well-represented, with a high number of studies for each link in the model.

2. Studies drawing samples from the United States (power distance score = 40) represented the largest proportion of studies in our dataset (43%). Thus, we also tested our models by splitting the power distance score at 40 such that “low power distance” group consisted of countries with power distance scores less than or equal to 40 (e.g., U.S.A., Canada, Germany; harmonic N = 3159), and “high power distance” group consisted of countries with power distance scores greater than 40 (e.g., China, Pakistan, France; harmonic N = 1167). The conclusions drawn from the results were identical to when we had split the power distance scores at 50.

3. Following the recommendation by Hunter and Schmidt (2004) to test for outliers, we conducted a series of analyses whereby we removed outliers based on effect size -/+2 *SD* from the mean (*k* = 10), sample size -/+2 *SD* from the mean (*k* = 11), and both effect size and sample size -/+2 *SD* from the mean (*k* = 21). The results and conclusion did not change.

4. We note that the direction of the relationships between interpersonal justice perceptions and CWB and between self-regulatory capacity impairment and CWB are contrary to what we predicted when relationships with the other mediators were taken into account. That is, *higher* justice was actually associated with *higher* levels of CWB, and higher self-regulatory capacity impairment was actually associated with *lower* levels of CWB. We provide our interpretation of these findings considering the rest of our results in the Discussion section.

5. Given we have available correlations for interpersonal justice perceptions for low power distance contexts, we ran an additional SEM model for low power distance subgroup with all four mediators. Result indicate that the mediated relationship via negative affect (indirect effect = .190, 95% CI [.166, .214]) is the strongest, and is significantly different from the mediated relationships via SERQ (indirect effect = −.002, 95% CI [−.014, .011]; indirect effect difference = .192, 95% CI [.165, .218]), via interpersonal justice perceptions (indirect effect = .058, 95% CI [.034, .083]; indirect effect difference = .132, 95% CI [.098, .166]), and via impaired self-regulatory capacity (indirect effect = −.035, 95% CI [−.050, −.020]; indirect effect difference = .225, 95% CI [.197, .253]).

6. We re-ran the moderator analysis with individualism/collectivism, and we found consistent results as when we ran the analyses with low/high power distance. As well, our results indicate that across cultures varying in individualism/collectivism, state negative affect remained the strongest mechanism in explaining the relationship between leader mistreatment and employee CWBs amongst the tested mechanisms, which is consistent with what we found when using power distance as a moderator. This is not surprising, given individualism/collectivism and low/high power distance were highly correlated in our data (*r* = −.87).

7. Although we also coded for CWBs directed at individuals (CWB-I), the measures used for CWB-I were often ambiguous as to specifically which individuals were targeted (e.g., measures of CWB-I may include supervisors). We therefore omitted CWB-I in our analyses.

8. We also ran two alternative models with only CWB-S or CWB-O included in the model. The results are largely similar to when we tested CWB-S and CWB-O simultaneously.

9. We thank an anonymous reviewer for suggesting this analytic approach.

## Appendix A. Search Terms.

[Fig fig5-15480518211066074]

*Note.* Although we were interested in interpersonal justice perceptions in our meta-analysis, we included all dimensions of justice in our search terms to cast a broad net.

## Appendix B. List of Journals Included in the Meta-Analysis.

[Fig fig6-15480518211066074]
